# Polymer-Coated Magnetite Nanoparticles for Protein Immobilization

**DOI:** 10.3390/ma14020248

**Published:** 2021-01-06

**Authors:** Kinga Mylkie, Pawel Nowak, Patryk Rybczynski, Marta Ziegler-Borowska

**Affiliations:** Faculty of Chemistry, Nicolaus Copernicus University in Torun, Gagarina 7, 87-100 Torun, Poland; kinga.mylkie@o2.pl (K.M.); nowak19981411@wp.pl (P.N.); pat_ryb@doktorant.umk.pl (P.R.)

**Keywords:** magnetite nanoparticles, magnetic nanoparticles, immobilization, protein, polymer coating

## Abstract

Since their discovery, magnetic nanoparticles (MNPs) have become materials with great potential, especially considering the applications of biomedical sciences. A series of works on the preparation, characterization, and application of MNPs has shown that the biological activity of such materials depends on their size, shape, core, and shell nature. Some of the most commonly used MNPs are those based on a magnetite core. On the other hand, synthetic biopolymers are used as a protective surface coating for these nanoparticles. This review describes the advances in the field of polymer-coated MNPs for protein immobilization over the past decade. General methods of MNP preparation and protein immobilization are presented. The most extensive section of this article discusses the latest work on the use of polymer-coated MNPs for the physical and chemical immobilization of three types of proteins: enzymes, antibodies, and serum proteins. Where possible, the effectiveness of the immobilization and the activity and use of the immobilized protein are reported. Finally, the information available in the peer-reviewed literature and the application perspectives for the MNP-immobilized protein systems are summarized as well.

## 1. Introduction

Nanotechnology development and the application of nanomaterials has been a very attractive topic for several years. Among synthesized and used nano-sized structures, magnetic nanoparticles (MNPs) are one of the most interesting and widely applied [1,2]. Such nanostructures are usually composed of a magnetic core and the surrounding shell. The core is mostly iron oxides, which can be in the form of magnetite (Fe_3_O_4_), hematite (α-Fe_2_O_3_), maghemite (γ-Fe_2_O_3_), and FeO [3,4,5,6]. However, of the mentioned iron oxides, magnetite (Fe_3_O_4_) is most often used as the core for MNPs preparation [7]. Apart from superparamagnetism, magnetite nanoparticles are characterized by many useful and unique properties such as high surface area, large surface-to-volume ratio, and easy separation under an external magnetic field. Due to this properties, they are widely used in bioseparation, catalysis, industrial processes and, above all, in biomedical sciences [8,9,10,11].

The biomedical applications of magnetite nanoparticles are primarily cancer diagnostics and therapies (Magnetic Resonance Imaging, Hyperthermia, Magnetic Field-Assisted Radiotherapy, Photodynamic Therapy), biocatalysis, pharmaceutical analysis, tissue engineering, biosensor, and the immobilization of biomolecules such as proteins [3,12,13,14,15]. However, pure, uncoated magnetite nanoparticles have some limitations in use by reason of the ability to spontaneously form aggregates (a result of the system’s desire to reduce surface energy, both under the influence of the magnetic field and the biological environment) [16]. Moreover, non-functionalized Fe_3_O_4_ nanoparticles are characterized by high chemical activity and susceptibility to oxidation, which often leads to a decrease or complete loss of magnetic properties. Covering the magnetite core with a coating, which may be inorganic and organic compounds, surfactants and polymers lead to the stabilization of the nanomagnetic material and, as a result, to the possibility of its wider use, e.g., in industry [17,18,19].

One of the most universal and widely used applications of magnetite nanoparticles is their treatment as carriers for protein immobilization [20,21]. The main problem in working with free, non-immobilized protein is the difficulty of its separation from the supernatant, which requires such techniques as ultrafiltration [22], ultracentrifugation, and microdialysis [23]. Protein immobilization on a magnetic carrier allows for a simple and quick separation of the nanoparticle–protein system from the supernatant by applying a simple magnet [20]. The basic condition that must be met by such a system is to maintain the activity of the immobilized protein. Moreover, the immobilization of a protein causes its structure to be targeted and, in some cases, this may facilitate e.g., the formation of an active protein–ligand complex. In the case of the immobilization of enzymes on the surface of magnetite nanoparticles, it is possible to reuse such a catalytic system, which is very beneficial [24,25,26,27].

As already mentioned, the surface of the magnetite core can be coated with various compounds, of which polymers seem to be the most attractive [12]. Deposition of the polymer on the surface of magnetite nanoparticles provides its chemical and thermal stability [16,28,29]. In addition, in the case of nanoparticles coated with polymers, a smaller tendency to their aggregation is observed, which significantly increases their usability [30,31]. Furthermore, most polymers, and in particular biopolymers, have in their structure reactive amino, carboxyl, and hydroxyl groups, which can be successfully used in the immobilization of proteins [30]. Therefore, in recent years, there has been a growing interest in the synthesis of magnetite nanoparticles coated with macromolecules and their subsequent application for the immobilization of bioligands [32,33,34].

The following review describes the utility of magnetite nanoparticles coated with polymers in protein immobilization. Both the cases in which the protein was immobilized by physical interactions (adsorption) and by covalent bonding with the polymer surrounding the magnetite core are discussed. Particular attention is paid to nanoparticles coated with natural polymers as those that find wider application in biomedicine as a result of their biocompatibility.

## 2. Synthesis of Magnetite Nanoparticles—Chemical Methods

**Figure 1:**
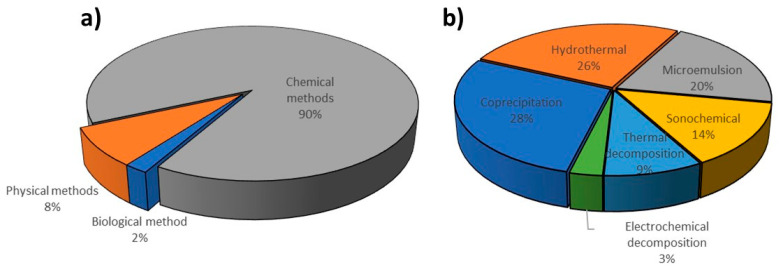
(**a**) A comparison of the synthesis of magnetic nanoparticles (MNPs) by three different routes; (**b**) classification of chemical methods for the synthesis of magnetic nanoparticles.

Synthetic methods for magnetic nanoparticles preparation can be classified according to the type of process that results in obtaining magnetic nanomaterial. Currently, the following synthesis approaches can be distinguished: chemical, physical, and biological. All of the listed methods have their advantages and disadvantages. Biological methods involving the use of microorganisms for the synthesis of nanoparticles allow for precise control of the obtained material. They are characterized by high process efficiency and very high repeatability. Moreover, they are inexpensive, but the fermentation process is quite time-consuming. Although physical methods for obtaining MNPs are easy to perform, it is difficult to control the size of the resulting nanoparticles in contrast to chemical methods covering about 90% of all techniques (Figure 1) [19]. 

The first literature reports of MNPs preparation appeared in the late 1960s in the works of Papell, who obtained nanoparticles by the physical method—grinding microcrystalline magnetite powder in the presence of solvents and surfactants in a ball mill. Unfortunately, MNPs obtained by this way were characterized by a high polydispersity index [35]. To prevent particle–particle agglomeration or sedimentation, Papell finally added oleic acid as a dispersing agent [36]. Subsequent works on this subject published by Sugimoto and Matijević concerned the chemical preparation of monodisperse magnetic nanomaterial of narrow size distribution, with mean diameters ranging between 0.03 and 1.1 μm. MNPs were obtained by FeSO_4_ interaction with potassium hydroxide in the presence of nitrate ion, and the resulting gelatinous suspension was kept at 90 °C for several hours [37]. In 2002, Sun and Zeng developed a synthesis of monodisperse magnetic nanoparticles with a small diameter of 4 nm. They used for this purpose the thermal decomposition of iron (III) acetylacetonate in phenyl ether in the presence of ethanol, oleic acid, and oleylamine [38]. Since then, new methods of preparation of MNPs with the desired sizes and properties are practically constantly being sought. 

Due to the fact that the methods of chemical synthesis of magnetic nanoparticles are the most commonly used in their preparation, only these methods are discussed in the next part of this review. 

### 2.1. Co-Precipitation Reaction

**Figure 2:**
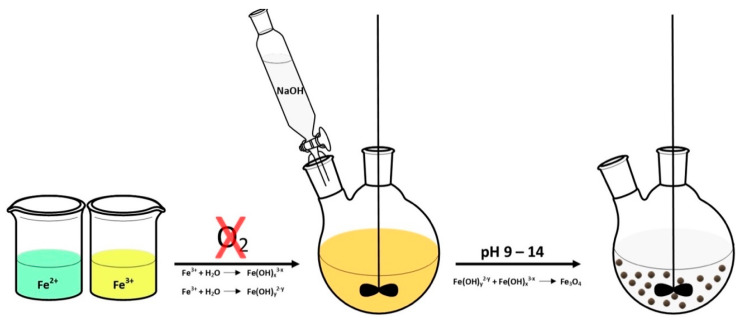
Synthesis of iron oxide nanoparticles by co-precipitation.

The co-precipitation reaction is the most popular method of magnetic nanomaterials synthesis. This route is widely used for the preparation of magnetite nanomaterials intended for biomedical applications due to non-toxic reagents. Co-precipitation is about reaction of magnetite formation in alkaline solution. It is necessary to keep anaerobic reaction conditions to prevent the conversion of magnetite to iron (III) oxide and then iron (III) hydroxide. Such conversion is very unfavorable for biomedical applications due to the ability of iron (III) oxide to form free radicals and the insolubility of iron (III) hydroxide. To avoid the oxidation of magnetite during the synthesis of nanoparticles, various types of stabilizers are introduced to protect the magnetite against oxygen. Moreover, magnetic phase and particle size can be controlled by changing the Fe^2+^/Fe^3+^ ions ratio, temperature, pH, and the type of used base solution [39,40]. The scheme of the magnetite synthesis from alkaline solutions of iron (II) and (III) salts is presented in Figure 2.

### 2.2. Thermal Decomposition 

**Figure 3:**
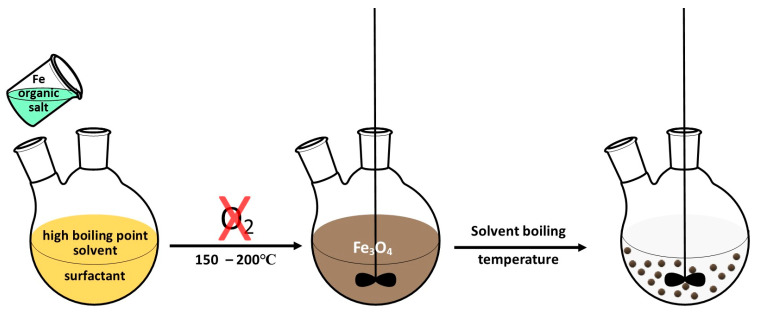
Synthesis of magnetite nanoparticles by thermal decomposition.

Thermal decomposition is based on the decomposition of metal precursors (e.g., acetylacetonates, carbonyls, or oleates) at high temperatures (150–300 °C) in the presence of organic solvents characterized with high boiling point (250–300 °C) such as octadecene or benzyl ether [41]. The presence of dispersants and hydrophobic ligands, including oleic acid, lauric acid, oleylamine, and hexadecyl amine, is required to control the size and shape of formed nanoparticles and prevent their aggregation [42,43]. This synthesis procedure is divided into three main steps. Firstly, a mixture of solvent with organometallic precursors, surfactants, and stabilizing agents is heated at a constant rate to reach the nucleation or decomposition temperature of the precursor. Next, the solution is heated to the boiling point of the solvent, which leads to the formation of small nanocrystals, and the final step consists of growth phase in which the solution is refluxed for some time and cooled to room temperature [44]. The schematic representation of this type of synthesis is presented in Figure 3.

However, the synthesis procedure becomes more complex, time-consuming, and more difficult to scale than the co-precipitation reaction; this method allows obtaining magnetite nanoparticles with very narrow size distribution and well-defined magnetic property [16]. Additionally, the appropriate proportions of the reagents and temperature control determine the synthesis of MNPs of defined shapes, sizes, and crystallinity. Unfortunately, there is often a lack of repeatability of the results obtained in this method due to the need to control many parameters during the process. Moreover, the MNPs prepared in this way are dispersible only in non-polar solvents (e.g., hexane) and are not miscible with water (as opposed to those obtained by co-precipitation), which can be a major limitation in some applications, especially biomedical [45].

### 2.3. Microemulsion Method

Microemulsions are thermodynamically stable colloidal suspensions in which, thanks to surfactants, two initially immiscible liquids coexist in one phase. In this technique, a stable dispersion of two immiscible solvents (water/oil) stabilized with a surfactant (e.g., dodecyl sulfate) is prepared [46]. Substrates for the production of nanoparticles are closed inside the micelle formed from water droplets surrounded by surfactant. The continuous phase in this case is the oil phase. The synthesis of MNPs takes place by introducing a second emulsion or a solution of a base into the system, which causes the rupture and coalescence of the micelles with the substrates, leading to the preparation of magnetite nanoparticles. The use of micelles allows for the stabilization of the system by limiting the nucleation, growth, and agglomeration of nanoparticles [47]. Obtained nanoparticles are separated from the emulsion by extraction with an organic solvent. The microemulsion method leads to nanoparticles with sizes from 1 to 100 nm. Compared to other methods, this procedure has several advantages, including the use of simple apparatus, the ability to synthesize many different materials with a high degree of control of particle size and composition, the preparation of nanoparticles with a crystalline structure and large specific surface area, and the use of simple synthesis conditions closed to ambient temperature and pressure [18]. Properties of magnetic nanomaterial prepared with the microemulsion method depend on the type and structure of the used surfactant [48].

### 2.4. Hydrothermal Method

**Figure 4:**
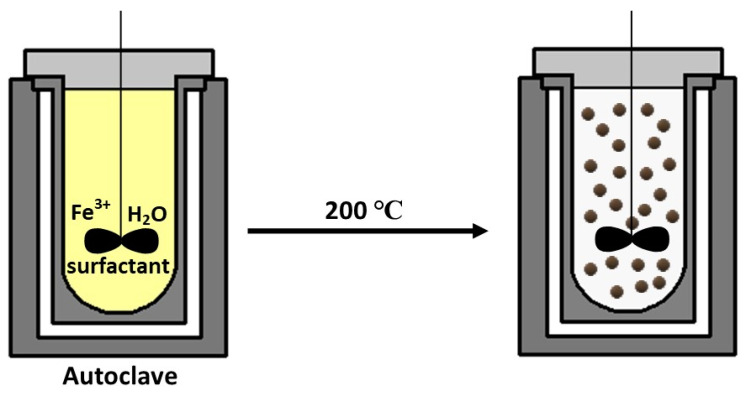
Schematic synthesis of magnetite nanoparticles by the hydrothermal method.

The hydrothermal method was first described by the German geologist Karl Emil von Schafhäutl in 1845 [45]. The general system is composed of (solid) metal linoleate, a liquid phase of ethanol–linoleic acid, and a hydrothermal (high temperature and high pressure) water–ethanol solution [18]. Typically, the reaction is carried out at about 220 °C, 13.79 MPa pressure for 72 h. [45,49]. As can be deduced from the high-pressure conditions, hydrothermal reactions are carried out using autoclaves or special reactors. For example, Wang, Zhuang, and Peng obtained monodispersed Fe_3_O_4_ nanoparticles with a size of 9 nm using a vigorously stirred mixture of FeCl_3_, ethylene glycol (high-boiling reducing agent), sodium acetate (electrostatic stabilizer), and polyethylene glycol as surfactant. The mixture was closed in an autoclave made of Teflon-coated stainless steel at a temperature of 200 °C up to 72 h (Figure 4) [50].

### 2.5. Sonochemical Processing

Sonochemical reactions involve the use of ultrasound to synthesize nanomaterials with a controlled size distribution using co-precipitation or decomposition reactions. [51]. This technique uses during synthesis the phenomenon of acoustic cavitation consisting of the propagation of sound waves in the range from 20 to 10 MHz. Sound waves cause pressure changes in the liquid layer close to the surface, as a result of which gas bubbles form. After placing the solution in an ultrasonic reactor of high temperature (>500 K), high pressure (>20 MPa), and high cooling rate (10^10^ Ks^−1^), nanoparticles are generated by the collapse of the bubbles [52,53]. These conditions are favorable for the production of highly monodisperse nanomaterials. This method has been used for preparation of several nanocomposites, and its versatility has been successfully confirmed in the synthesis of magnetite nanoparticles [54]. Vijayakumar described the sonochemical synthesis of pure Fe_3_O_4_ powder, which is a monodisperse material with a particle size of 10 nm, minimizing the nanoparticle agglomeration process [55].

### 2.6. Electrochemical Methods

The literature describes many methods of electrochemical preparation of magnetic nanoparticles [4,56,57,58,59,60,61,62,63]. Moreover, a lot of articles indicate a significant impact of the density (J) and potential (E) of used redox systems on the prepared MNPs size as well as the possibility of its control [57,58,61,62,63]. In one of them, magnetite nanoparticles, ranging in size from 20 to 30 nm, were produced by electro-oxidation using iron as an electrode material: anode and cathode. The required distance between the two electrodes was about 1 cm. Tetramethylammonium chloride ((CH_3_)_4_NCl) was added to the electrolyte solution as a surfactant, and the reaction was carried out in 30 min at relatively low temperature (6 °C) [57]. This reaction conditions lead to magnetite nanoparticles homogeneous in size and spherical in shape. Subsequently, another electrochemical method was proposed with iron as the anode and water as the electrolyte without any surfactant addition [61]. In this procedure, a change in the distance between the electrodes (from 2 to 6 cm) and the current density from 205 to 415 μA/cm^2^ was used for the size of the resulting nanoparticles regulation. Moreover, it was noticed that the particle size (from 10 to 30 nm) increased simultaneously with the increase of the current density and decrease of the distance between the electrodes [61]. Marques et al. proposed the electro precipitation of magnetic nanoparticles in ethanol solution with iron (III) nitrate nonahydrate (Fe(NO_3_)_3_ × 9H_2_O) as an iron precursor, and the precipitation of Fe(OH)_3_, which was then reduced to magnetite in the presence of hydroxyl ions formed at the cathode [62]. This route enables the production of superparamagnetic magnetite nanoparticles with controlled size and dispersion [62].

Summarizing, Table 1 shows a comparison of the advantages and disadvantages of the chemical methods of magnetite nanoparticles preparation that were discussed above.

## 3. Modification of Bare Magnetite Nanoparticles

**Figure 5:**
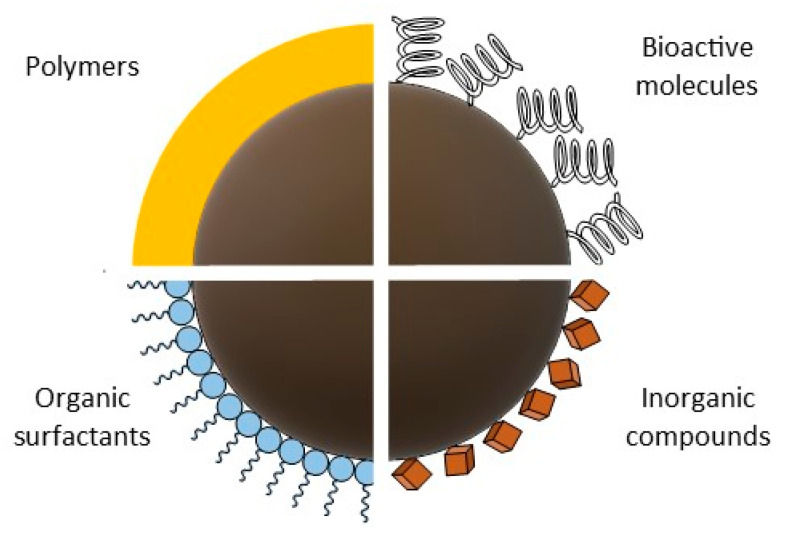
Schematic illustration of the main shells for functionalization of iron oxide magnetic nanoparticles (MNPs).

Bare magnetite nanoparticles without surface modification with low- or high-molecular compounds have a limited applications as a result of the spontaneous aggregates formation [65]. In addition, uncoated MNPs are characterized by many unfavorable properties, such as chemical instability, poor biodegradability in the physiological environment, and non-specific interaction with blood serum proteins [66]. Moreover, the lack of a stabilizing coating for the magnetic core makes magnetite susceptible to oxidation with oxygen in the air, which as already mentioned is particularly negative for biomedical applications. The cover layer also reduce the risk of adverse effects of material when nanoparticles are used as therapeutic agent [67,68] and allows for the improvement of nanoparticle dispersion in solutions, limiting their toxicity, as well as improving the physicochemical and functional properties. The shell covering the magnetic core can be generated in situ during the preparation of the nanoparticles themselves, or then, the previously obtained pure nanoparticles can be coated. Organic surfactants and inorganic compounds, bioactive compounds, as well as natural and synthetic polymers are used for surface of nanoparticles coating (Figure 5) [69].

### 3.1. Organic Surfactants

**Figure 6:**
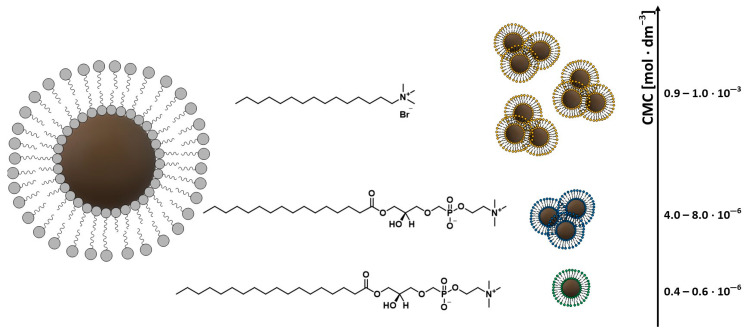
Schematic representation of MNPs and surfactant structures. The clustering tendency is represented as function of values of the critical concentrations.

Surfactants such as oleic acid, lauric acid, alkylsulfonic, and alkylphosphonic acids are used for magnetic nanoparticles stabilization [20]. Sahoo et al. proved based on photos from an electron microscope that carboxylate surfactants provide particles with better isolation and dispersibility than phosphonate surfactants [70]. Unfortunately, long hydrocarbon chains from the surfactant structure cause the nanoparticles to be highly hydrophobic, which makes their use in vivo much more difficult [71]. The research of Luchini and co-authors was aimed at overcoming these limitations. They showed that the use of surfactants with low critical micelle concentration (CMC) values for the functionalization of MNPs resulted in their greater dispersion in solutions, and additionally, the coating process of nanoparticles was more efficient than with surfactants with higher CMC values (Figure 6) [72].

### 3.2. Inorganic Compounds

**Figure 7:**
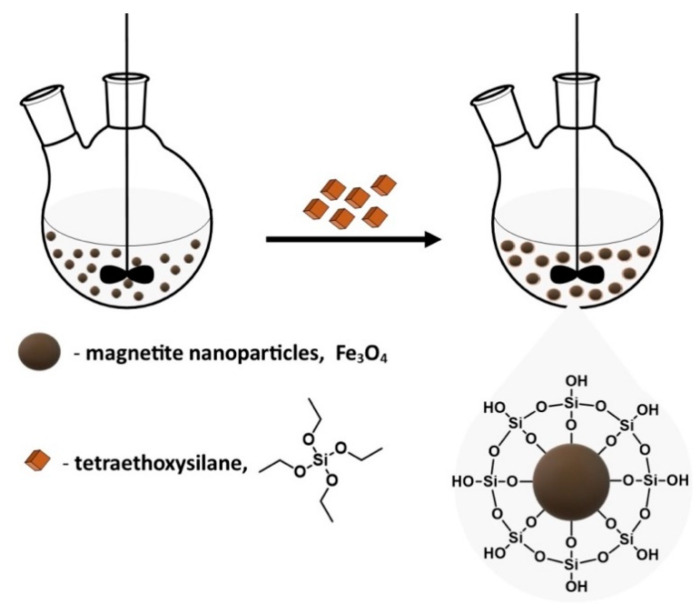
Functionalization of the surface of MNPs using tetraethoxysilane (TEOS).

Coating the magnetite core with inorganic compounds, apart from ensuring the stability of nanoparticles, quite significantly broadens the area of their application [73]. The most commonly used compounds for this purpose are silicon compounds, metals and their oxides, sulfides, carbon in the form of graphene and its oxide, and nanotubes [74,75,76]. Silica is a classic coating material for nanoparticles, as it can enhance their dispersion in aqueous solutions, endows them greater durability, and protects them in acidic environments [69,77,78]. The coating of nanoparticles with silica (SiO_2_) was carried out based on a modified Stöber process [79]. This method uses tetraethoxysilane (TEOS) as a silicon shell precursor in the hydrolysis and condensation reaction (Figure 7). The synthesis is carried out in an aqueous ethanol solution in the presence of ammonia as a homogeneous catalyst [79]. The presence of silanol groups on the surface of magnetic nanoparticles reduces their potential toxicity and also causes the colloidal stability of nanoparticles in the physiological pH range. Thanks to that, MNPs can be successfully used in molecular biology and medicine [80,81,82].

Another example of a silicon compound used for magnetite core stabilization is aminosilane (SiO_2_-NH_2_). This compound due to the presence of the basic amino group is positively charged at a pH value of about 7.4. Since plasma membranes have huge negatively charged domains, it has been proved that cationic aminosilan easily penetrates inside the cells [83,84]. 

Carbon-based materials as an inorganic compound are also applied to MNPs surface coatings to enhance their stability, biocompatibility, and dispersity. Carbon-coated magnetite nanocomposites have found wide application as catalysts, electrode supercapacitors, microwave absorbers, and anode materials for lithium-ion batteries [74,75,76]. 

Noble metals such as gold and silver, which are characterized with biocompatibility and resistant to chemical reactions such as oxidation and corrosion, were usually used for MNPs stabilization [85]. The surface of the nanoparticles covered with these metals characterizes not only the stability under physiological conditions, but also, its ligand binding ability would be enhanced and also the formation of harmful free radicals could be prevented [86]. 

Increasingly, metal oxides such as TiO_2_ [87,88,89], SnO_2_ [73,90]_,_ Cu_2_O [91], ZnO [92], CdS [93], ZnS [94], PbS [95], Bi_2_S_3_ [96], or sulfides have been also used for the protection or functionalization of MNPs. For example, Saffari et al. prepared superparamagnetic Fe_3_O_4_-ZnO nanocomposites with 10% ZnO content by adopting the sonochemical method. It was reported that the Fe_3_O_4_/ZnO nanocomposite has excellent photocatalytic properties. [92].

### 3.3. Polymers and Bioactive Molecules

Biological active compounds such as lipids, peptides, and proteins are used for magnetic nanoparticles coating without loss of material magnetization [73,80,97,98]. Jahanban-Esfahlan and co-authors deposited human (HSA) and bovine (BSA) serum albumin on magnetic nanoparticles surface via desolvation [99]. Such nanoparticles have a negatively charged surface that prevents electrostatic interactions with negative biological elements such as plasma proteins and blood cells, thus preserving the stability of nanoparticles [100]. Nosrati used a magnetic nanoparticles coated with BSA prepared by chemical desolvation and co-precipitation as curcumin carriers [100].

Polymers are widely used as a shell covered the surfaces of nanomaterials. They can prevent MNPs oxidation and give nanoparticles collateral stability [101]. Several approaches have been developed for MNPs functionalization with polymers, where the common methods include in situ and post-synthesis coating [73]. In the in situ approach, the conventional routes are mini/micro-emulsion polymerization and the sol–gel process, while the post-synthesis coating method is carried out as a result of chemical reactions or by non-covalent interactions of the polymer with the magnetite core. [73]. The nature of the polymer may be synthetic: polyethylene glycol, polyacrylic acid, poly (vinylpyrrolidone), poly (vinyl alcohol), poly (methacrylic acid) or natural, as in the case of chitosan, starch, cellulose, agarose, and dextran. 

## 4. Protein Immobilization Methods

Protein immobilization is a biotechnological technique where protein is fixed in a suitable matrix that restricts its movement to increase stability and, in the case of an enzyme protein, to allow its reuse with maintaining immobilized protein activity [102]. The first literature reports on the immobilization of catalytic proteins come from 1916 when Griffin and co-authors adsorbed the enzyme invertase on a solid matrix of charcoal and aluminum hydroxide [103]. 

Choosing the right immobilization technique for a given protein plays a very important role. The cost of immobilization, the possible inactivation of the protein, the toxicity of the reagents, and the properties of the system obtained in this way should be taken into account [104]. Protein immobilization methods can be divided into two categories: chemical and physical. The chemical method uses the formation of new covalent or ionic bonds, and the physical method mainly involves hydrophobic or van der Waals interactions between the protein and the support. While chemical immobilization creates strong bonds between the molecule and the carrier, this results in greater durability of the immobilization compared to physical immobilization but may cause changes in the structure of the immobilized molecule. The most commonly used techniques for the immobilization of proteins, including enzymatic proteins, are adsorption, trapping, covalent bonding, and cross-linking. As a rule, immobilized proteins are less sensitive to changes in pH, temperature, and the action of toxins. Due to the possibility of changing the protein structure, immobilization is not a flawless method. For example, carrying out the immobilization process may reduce the catalytic properties of the enzyme. Factors that may affect the activity of the immobilized protein are the stiffening of the protein structure and diffusion resistance in the free transport of ligands to and from the active center of the protein [105].

There are some parameters describing the performed immobilization such as the yield of immobilization (protein loading), and in the case of enzymatic protein, efficiency and activity recovery [106].

In processes using proteins such as analytics, bioseparation, and catalysis, separation of the used protein from the supernatant is a key parameter prompting the use of immobilization. As already mentioned, the immobilization of the protein on the surface of magnetic nanoparticles (MNPs) primarily ensures easy separation of the carrier–protein system using an external magnetic field. In recent years, MNPs are one of the most frequently used supports for this purpose because of their large surface area and easy functionalization. The main advantage of using MNPs as carriers for the immobilization of e.g., enzymatic proteins is the possibility of reusing the biocatalyst after separation from the reaction medium. Immobilization on a magnetic carrier is mainly physical immobilization and covalent bonding on the surface of the support. Due to the fact that the immobilization takes place on the surface of a permanent support, physical adsorption and covalent binding are usually applied [104,107,108,109]. 

### 4.1. Adsorption of Protein on MNPs Surface

As it was discussed, the adsorption of proteins on solid carriers is one of the oldest and simplest physical immobilization techniques. The protein can be immobilized by mixing with an appropriate adsorbent. It is important to ensure the right immobilization conditions: pH and ionic strength of the solution. Only weak interactions, such as hydrophobic and van der Waals interactions and hydrogen bonds, keep the protein molecule on the surface of the carrier. In general, enzyme immobilization through the technique of physical adsorption is quite simple and may have a high commercial potential due to its simplicity, low cost, and retaining high enzyme activity as well as a relatively chemical-free biomolecules binding. Naturally, this method suffers from several disadvantages such as low resistance to changes in pH, temperature, and the ionic strength of the buffer. Furthermore, the physical interaction is generally too weak to hold the protein bound to the carrier, which may cause the protein desorption from the carrier surface in solution [109,110,111]. Desorption usually leads to a loss of protein activity and contamination of the supernatant with protein, which may prevent the reuse of immobilized proteins, especially for analytical applications. Additionally, protein adsorption on the carrier surface often leads to conformational changes in its structure and loss of protein activity. This immobilization technique allows the use of many carriers, because the most important part of this method is the appropriately high affinity of the carrier to the immobilized protein. However, depending on the type of used carrier, the amount of deposited protein may be different, and the effectiveness of immobilization depends not only on the type of support but also on the used enzyme and the immobilization conditions.

### 4.2. Covalent Binding of Protein on MNPs Surface

Covalent immobilization is a chemical method of protein binding on the carrier surface. It involves the formation of a covalent bond between the functional groups of the protein and support. It is advisable to use such protein functional groups in the immobilization process, which are susceptible to chemical modification and do not participate in the stabilization of the third and fourth-order structure of the protein [104]. The cysteine thiol group, phenyl ring of tyrosine, imidazole group of histidine, and amino group of lysine are most often used for this purpose. In order to easily react the above-mentioned functional groups of the protein, the surface of the support should contain reactive amino, hydroxyl, carboxyl, vinyl sulfone, vinyl ketone, oxirane, aldehyde, halide, and thiol groups. Depending on the type and presence of functional groups on the carrier surface, the formation of the carrier–protein covalent bond consists in the following reactions: arylation, amidation, diazotization, alkylation, and the formation of Schiff bases or amide bonds. Coupling agents such as glutaraldehyde (GA) [112], glyoxal [4], epichlorohydrin [104], 1-ethyl-3-(3-dimethyl-aminopropyl) carbodiimide (EDC), and *N*-hydroxysuccinimide (NHS) [113] are often used in protein covalent immobilization [114]. For example, EDC activates carboxyl groups of protein and forms an amine reactive O-acylisourea intermediate that spontaneously reacts with primary amines to form an amide bond and an isourea by-product. The O-acylisourea intermediate is unstable in aqueous solutions, and failure to react with an amine will cause hydrolysis of the intermediate, regeneration of the carboxyls, and the release of an *N*-substituted urea [115,116]. Therefore, it is necessary to quench the EDC activation reaction with a thiol-containing compound such as 2-mercaptoethanol. EDC couples NHS to carboxyls, which forms an NHS ester that is considerably more stable than the O-acylisourea intermediate and allows for efficient conjugation to primary amines at physiological pH. The advantageous quality of EDC is that it is water soluble and dissolves in aqueous buffer solutions, similar to most biological macromolecules [117]

The efficiency and effectiveness of immobilization depends, inter alia, on the number of these groups present on the surface of the support. Naturally, the immobilization efficiency increases with the increase in the number of functional groups on the carrier surface that are available for protein binding. However, in the case of the enzyme protein, too much “packing” of the enzyme on the support surface may lead to decrease in enzymatic activity or inactivation [106].

The number of carriers that can be used in this immobilization technique is less than in physical immobilization. The support surface can be covered with inorganic and organic compounds as well as composites and polymers, and the most important criterion when selecting a carrier is the presence of reactive functional groups on its surface that are able to form covalent bonds.

As it can be seen, each of the methods has its advantages and disadvantages, which are briefly summarized in Table 2.

## 5. Immobilization of Proteins on Polymer-Coated Nanoparticles 

As a result of susceptibility to modification, thermal stability, and resistance to pH changes, as well as mechanical properties, polymers are very often used for a magnetite nanoparticles core coating. As it is known in view of the structure and source of obtaining the polymer, we can divide them into synthetic and natural polymers (biopolymers). Consequently, in this article, polymer-coated nanoparticles used for protein immobilization were divided based on this division.

### 5.1. Immobilization of Proteins on Nanoparticles Coated with Synthetic Polymers

Synthetic polymers, in contrast to natural macromolecules, are prepared from the corresponding monomers by chemical synthesis. These polymers are characterized by high purity and their weight and composition are controlled in the synthesis process. The purity of the material is of particular importance for their use in biomedicine and e.g., pharmaceutical formulation [120]. The basic methods of obtaining synthetic polymers are generally bulk, solution, suspension, or emulsion technology using homogeneous or heterogeneous catalysts of acid, alkali, or radical species or transition and rare metal catalysts. Moreover, synthetic polymers are versatile materials that can be processed into biomedical foams with a wide range of mechanical, thermal, and degradation properties. The tailoring of these properties can be achieved by using different polymeric families such as polyesters, polyurethanes, and tyrosine-derived polymers. One of the parameters determining the final properties of a polymer is also its porosity [121]. The applications of magnetite nanoparticles coated with synthetic polymers in protein immobilization are presented below. 

#### 5.1.1. Immobilization of Proteins on Nanoparticles Coated with Polyethylene Glycol (PEG)

Polyethylene glycol (PEG) is hydrophilic, uncharged, and non-immunogenic linear polyether macromolecule. As a result of its low toxicity and ease of excretion from the body through the kidneys (for PEGs less than 30 kDa) and in feces (for PEGs > 20 kDa), PEG is also very often used for biomedical and therapeutic applications [122]. In addition, of all the synthetic polymers, this macromolecule is the most widely used for magnetic core coating, especially to ensure the high colloidal stability of the nanomaterial [123]. However, in the literature, there are only a few reports on the use of PEG-coated magnetite nanoparticles as a carrier for proteins.

**Figure 8:**
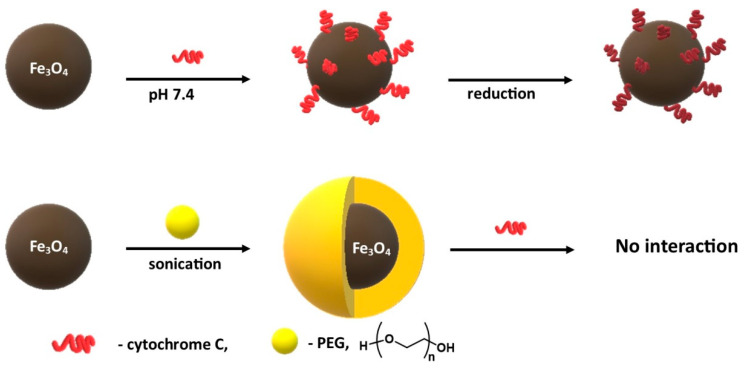
Schematic representation of the interaction of Cytochrome C with the bare and polyethylene glycol (PEG)-coated Fe_3_O_4_ MNPs.

On the basis of Mukhopadhyay’s [124] research, it can be concluded that PEG coatings are effective means of protecting biomolecules against the toxicity generated by magnetite nanoparticles. Mukhopadhyay [124] et al. described the interaction of magnetic nanoparticles coated with ethylene glycol and Cytochrome C, which acts as an electron transporter in the respiratory chain. The interaction of naked uncoated nanoparticles with Cytochrome C led to the reduction of this protein, while the magnetite nanoparticles coated with PEG showed no affinity for Cytochrome C and finally did not reduce it (Figure 8). 

Chang and co-authors successfully applied magnetite nanoparticles coated with polyethylene glycol (PEG) to the novel method of the exosome purification. For this purpose, they used nanoparticles obtained by chemical co-precipitation reaction. Next, this material was used to remove fetal bovine serum (FBS) from a biological fluid by means of physical immobilization [125]. Exosomes are secreted e.g., by cancer cells that are responsible for metastasis and subsequent cancer growth. The detection of exosomes is a key step in the early cancer diagnosis [126,127]. Exosomes exist in a biological fluid such as blood that also contains proteins, so it is important to remove proteins from the biological fluid before exome detection to avoid test interference. The research results indicate that the proposed method of using PEG-coated magnetite nanoparticles for protein removal and exosome purification is quick and simple. 

#### 5.1.2. Immobilization of Proteins on Nanoparticles Coated with Polyvinyl Alcohol (PVA)

Polyvinyl alcohol (PVA) is hygroscopic, colorless, and odourless biocompatible and biodegradable synthetic macromolecule [128]. One of the most important properties of PVA is the ability to form multiple hydrogen bonds between the polymer chains, which prevents aggregation and agglomeration of magnetic nanoparticles coated with this polymer [80,129,130]. 

Mahmoudi [131] et al. published the physical immobilization of human protein—transferrin on magnetite nanoparticles, both naked and coated with PVA. In addition, it was also the first article to describe the irreversible conformational changes of a specific protein as a result of interaction with MNPs. After the adsorption of transferrin on the nanoparticles surface, the main function of the protein, which is the transport of iron between cells, was changed. The changes that took place in the structure and activity of transferrin were irreversible. The activity of the desorbed protein was tested, and transferrin was found to be inactive. 

Laochai [132] and co-authors published the synthesis of magnetite nanoparticles coated with a mixture of polyvinyl alcohol and chitosan (CS). Bare magnetite nanoparticles were synthesized by a simple method of co-precipitation and in the next stage coated with PVA and s chitosan (CS) layer. Then, the horseradish peroxidase was immobilized on the surface of prepared nanoparticles. This enzyme is usually used for hydrogen peroxide detection via catalytical oxidation of the substrate of hydrogen peroxide and o-dianazidine. A colored product of this reaction is formed whose concentration is proportional to the concentration of enzyme and measured [133]. It was also proved that peroxidase immobilized on PVA/CS-coated magnetic nanoparticles retained its activity in ten catalytic cycles. 

Sahin and co-authors published the synthesis of magnetic nanoparticles coated with polyvinyl alcohol and trypsin covalent immobilization on their surface with glutaraldehyde as a linker [134]. Trypsin is hydrolase that selectively catalyzes the hydrolysis of peptide bonds [135]. According to the published results, immobilized trypsin showed at 40 °C greater stability than the free enzyme. Additionally, after 12 days of storage at 4 °C, immobilized enzyme retained about 50% of its initial activity, while the activity of free trypsin stored under the same conditions was only 19%. In addition, the reuse of immobilized enzyme was also tested. After eight catalytic cycles, the trypsin deposited on the MNPs retained 56% of the original system activity. The efficacy of the immobilized trypsin was assessed in a study based on the digestibility of Cytochrome C. Immobilized trypsin showed effective proteolytic activity in a shorter time (15 min) than free trypsin (24 h). 

#### 5.1.3. Immobilization of Proteins on Nanoparticles Coated with poly(D,L-lactide-co-glycolide) (PLGA)

Poly(D,L-lactide-*co*-glycolide) is a copolymer synthesized by a random ring-opening copolymerization of two different monomers, cyclic dimers of glycolic acid (1,4-dioxane-2,5-diones) and lactic acid [136]. It shows great potential in drug transport and tissue engineering due to its biocompatibility and biodegradability [137,138], and it is soluble in most commonly used solvents. Depending on the proportion of each monomer in the copolymer structure and polymer molecular weight, PLGA shows different properties. Usually, higher amounts of lactide in PLGA lead to less hydrophilic material with slow degradation as a result of lower water absorption [69].

PLGA-coated magnetite nanoparticles have many advantages in drug delivery, especially in drugs protection against degradation. Moreover, they can also improve the pharmacokinetic and pharmacodynamic profiles of transported drugs. Another important advantage of PLGA over other polymers is that this macromolecule is approved by the FDA (U.S. Food and Drug Administration) and EMA (European Medicines Agency) in various drug delivery systems, which means that PLGA-coated nanoparticles can be used in clinical trials in a shorter period of time [139,140]. One of the most interesting applications of MNPs in biomedicine is their design to have additional fluorescent or luminescent properties. Such structures can be obtained by chemical or physical modification of the polymer shell surrounding the magnetite core [141,142]. This multimodal approach ensures the specific recognition and attachment of MNPs to the target cell, drug delivery, and the possibility of in vitro and in vivo bioimaging using optical methods, tomography, or magnetic resonance. 

**Figure 9:**
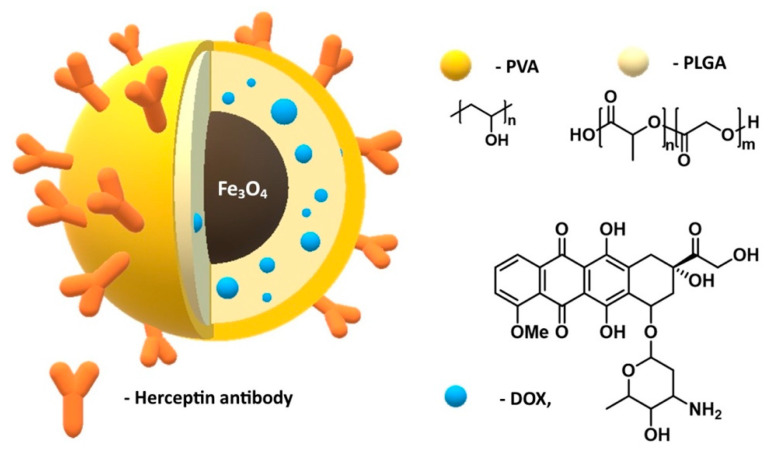
Schematic illustration of magnetic polyoly(D,L-lactide-co-glycolide) (PLGA)/polyvinyl alcohol (PVA)/doxorubicin (DOX) nanoparticles for diagnosis and treatment of cancer.

Jaemoon Yang and co-authors synthesized a multimodal nanocomposite using inorganic and organic materials for cancer detection and treatment [143]. The nanoemulsion method was used to incorporate doxorubicin, an anti-cancer drug (DOX), into magnetic nanoparticles coated with a mixture of poly (D,L-lactide-co-glycolide) and PVA (Figure 9) [144]. The Herceptin (HER) antibody used in the treatment of breast cancer was also immobilized on the surface of these nanoparticles (with EDC/NHS as a linker) without loss of its affinity for cancer cells. Additionally, the drug enclosed in the polymer shell was released in a balanced manner without any inhibition. These multi-functional composites may have applications in targeted drug delivery, MRI probes, and also cell separation.

**Figure 10:**
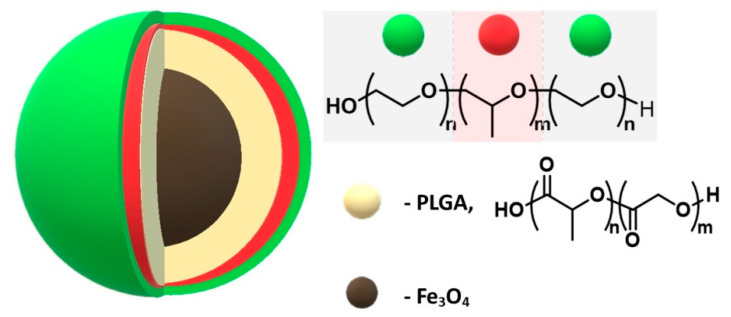
Scheme structure of poloxamer and adsorption of poloxamer onto MNPs-coated PLGA.

Shubhra and co-authors published a modification of the surface of magnetite nanoparticles coated with poly (D,L-lactide-co-glycolide) with a non-ionic copolymer—poloxamer (Pluronic F68, PF68) for the immobilization of bovine serum albumin (BSA) [145]. The protein adsorption capacity of this material was compared with poly(D,L-lactide-*co*-glycolide)-coated nanoparticles (Figure 10). The UV-Vis spectrophotometric analysis showed that the BSA adsorption on PF68-modified nanoparticles was reduced by about 50% in relation to poly (D,L-lactide-co-glycolide) modified nanoparticles. 

#### 5.1.4. Immobilization of Proteins on Nanoparticles Coated with Polyethyleneimine (PEI)

Linear polyethyleneimines contain only secondary amines as opposed to branched PEI, which contains primary, secondary, and tertiary amine groups. There are two carbon atoms in each PEI molecule with one protonated nitrogen atom. Due to the different pK_a_ values of the primary, secondary, and tertiary amine groups, PEI can scavenge protons under different pH conditions, which is known as the “proton sponge” mechanism. PEI was developed to condense DNA through the electrostatic interaction between the positive and negative charges of the DNA phosphate group [146,147]. A quaternary ammonium derivative of branched polyethyleneimine (bPEI-met) has also been synthesized, and it was found that it exhibits antibacterial activity by disrupting bacterial cell membranes [148]. Due to its unique properties, PEI appears to be one of the most suitable molecules for the surface modification of MNPs for biomedical applications.

**Figure 11:**
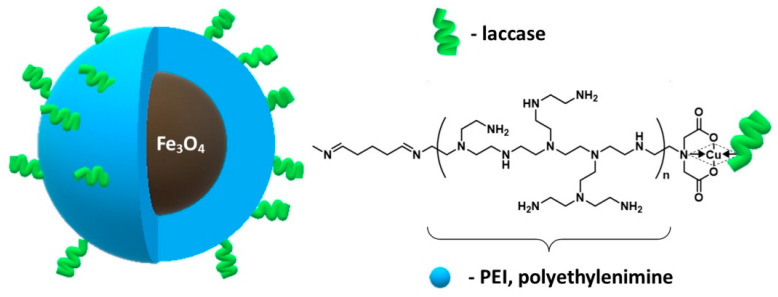
Scheme polyethylenimine-modified Fe_3_O_4_ nanoparticles (Fe_3_O_4_–NH_2_–PEI NPs) structure with chelated Cu^2+^ and immobilize laccase.

In 2016, Xia and co-authors described the synthesis of polyethyleneimine-coated nanoparticles (Fe_3_O_4_–NH_2_–PEI MNPs) [149]. Afterwards, this nanomaterial was chelated with copper ions in order to immobilize the *Trametes versicolor laccase* by physical immobilization—adsorption (Figure 11). Laccase as a multi-copper oxidase (belongs to the group of polyphenol oxidases) can be produced by numerous plants, funguses, and bacteria [150,151]. As a result of its relatively low substrate specificity and high catalytic activity, it has gained extensive attention in various fields such as environmental remediation, the pulp and paper industry, and biosensing [152]. However, the industrial applications of this enzyme are limited due to the low stability and poor reusability of free laccase [150,153]. The results obtained for the enzyme immobilized on (Fe_3_O_4_–NH_2_–PEI MNPs) nanoparticles were compared with those obtained for the enzyme immobilized on the particles without a polymer coating (Fe_3_O_4_–NH_2_ MNPs) containing only amino groups. It was noticed that polymer-modified nanoparticles show a higher adsorption capacity compared to nanoparticles without the polymer coating. Additionally, the recovery of laccase activity for nanoparticles with polyethyleneimine was two times higher than for magnetite nanoparticles without polymer. In addition, the activity of the immobilized enzyme improved significantly; the specific laccase activity was 101.33 times higher than for free enzyme. Moreover, immobilization allowed the enzyme to be reused. Immobilized enzymes on PEI-coated nanoparticles preserved 44.89% of their original activity after the 5th reuse. The activity loss in these steps may be related to particle agglomeration and the inactivation of laccase upon use.

Interesting research with polyethyleneimine-coated magnetic nanoparticles in gene therapy—magnetofection was published by Zuvin et al. in 2019 [154]. In this study, a new magnetic trigger system consisting of four rare earth magnets on a rotating table for a better magnetofection effect was designed and manufactured. Magnetic nanoparticles coated with polyethyleneimine with a green fluorescent protein (GFP) carrying DNA were used as model material. Magnetofection has been tested on the breast cancer cell line (MCF7). The results showed that the magnetic field exposure increased the transfection efficiency. 

It can be seen that polymers have different protein adsorption capacity resulting from the presence of different functional groups, different arrangements, and different molecular weight. Wiogo et al. performed the co-precipitation synthesis of bare magnetic nanoparticles that were in the next step modified by sonication with linear polymethacrylic acid (20 kDa), linear and branched polyethyleneamine (25 kDa), and branched oligoethyleneimine (800 Da) [155]. Next, the adsorption capacity of each material was tested using the biological serum proteins (fatal bovine serum). Based on the obtained results, it can be concluded that nanoparticles coated with branched polyethyleneimine adsorb the largest amount of serum protein, while nanoparticles coated with linear polymethacrylic acid showed the lowest interaction with proteins. The differences in the amount of immobilized protein resulted from the conformation of the polymer on the surface of the MNPs. The results of the interaction of nanoparticles obtained by Wiogo et al. with serum proteins are presented in the Table 3.

It was shown that the design of nanoparticles for biomedical applications can be improved through the appropriate selection of functionalization polymers and understanding the factors governing the stabilization mechanism.

Kannan et al. reported the immobilization of two lipases: *Candida rugosa* and *Mucor miehei* on polyethyleneimine (PEI)-coated MNPs [156]. These nanoparticles were used in chromatography as an anion exchanger for lipase separation, which resulted in retaining a significant part of the enzyme on the support. Moreover, PVP (polyvinylpyrrolidone) has been used in this research to reduce the amount of protein bound to the filling. The PEI-coated Fe_3_O_4_ nanoparticles were further coated with (1.0–2.5%) PVP solution at about 4 °C with 200 rpm for overnight to (1.0–2.5%) PVP-PEI-Fe_3_O_4_. Shielding with 2% PVP improved the elution of lipases with 1 M NaCl as eluent. The elution of *Mucor miehei* lipase increased from 56.8% to 68.3%. Similar results were observed for lipase from *Candida rugosa*. 

Gräfe et al. [157] and Calatayud et al. [158] published the formation of the protein corona on PEI-coated magnetic nanoparticles. Materials designed for biomedical and therapeutic applications generally come into contact with protein reach body fluids. Proteins are usually adsorbed on the surface of material to form an enveloping layer known as the “protein corona”. This process can be thought of as physical immobilization. The protein corona, which is formed as soon as nanoparticles come into contact with biological systems, plays a key role in the biological role of nanoparticles. Gräfe et al. describe a strategy to control the amount of adsorbed proteins on the surface of nanoparticles and the effect of such corona proteins on particle–cell interactions. Polyethyleneimine (PEI)-coated magnetic nanoparticles (MNPs) were incubated in a medium consisting of fetal calf serum (FCS) and the nutrient broth used for cell culture. As it can be expected, during the incubation process, the surface of PEI-modified magnetic nanoparticles was covered with serum proteins by physical immobilization. Next, the human HBMEC (Human Brain Microvascular Endothelial Cells) line was used to study the interaction with nanoparticles. The results show that the presence of the corona reduces the interaction of the nanoparticles with HBMEC during short-term incubation depending on FCS concentration [157].

**Figure 12:**
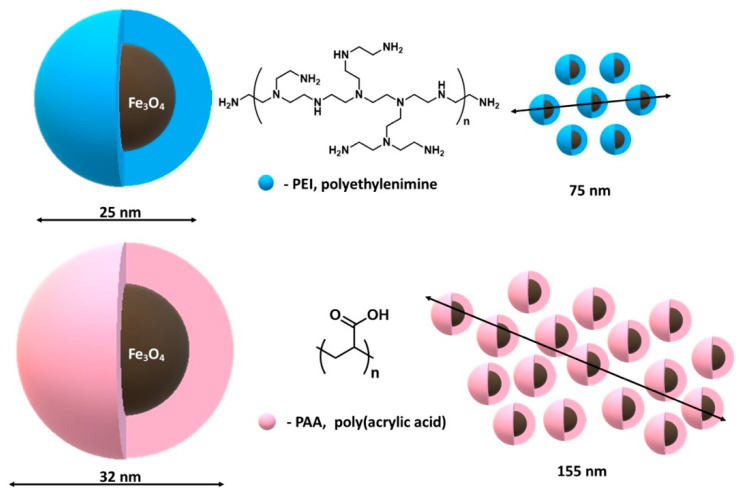
Sketched evolution of the particle agglomeration process for the MNPs when in their as-prepared suspension in water.

On the other hand, Calatayud et al. [158] focused on the process of protein adsorption on magnetic nanoparticles functionalized with polyethyleneimine and poly (acrylic acid) after immersing them in a cell culture medium (Figure 12). It was noticed that after 24-h incubation, large aggregates of proteins are formed on MNPs with a hydrodynamic size of 1500 nm (for nanoparticles coated with poly aryl acid) and 3000 nm (for nanoparticles coated with polyethyleneimine) [158]. 

The study presents the effect of the produced protein clusters properties on the absorption of SH-SY5Y cells. Despite the negative z-potential with similar values for both MNPs in cell culture, it was observed that PEI-MNPs are incorporated in in much greater amounts than polyacrylic acid (PAA)-MNP units. Quantitative analysis showed that SH-SY5Y cells can incorporate 100% of the added PEI-MNPs up to 100 pg/cell, whereas for PAA-MNPs, the uptake was less than 50%. This result suggests the possibility of controlling non-specific protein adsorption onto MNPs by proper functionalization of their surface. The impact of the final properties of these clusters on the cell uptake, which is typified by the much larger mass of attached PEI-MNPs compared to the PAA-MNPs, was demonstrated.

#### 5.1.5. Immobilization of Proteins on Nanoparticles Coated with Polyacrylic acid (PAA)

Polyacrylic acid (PAA) is a non-toxic polyanion (in which each unit has a carboxyl group), synthetic, high molecular weight polymer [159]. PAA is a weak polyelectrolyte with a degree of dissociation depending on the pH of the solution and its ionic strength. Moreover, it is a water-soluble macromolecule with a high density of reactive functional groups. These properties provide strong connections between iron oxide and biomolecules, which makes PAA-coated magnetite nanoparticles very attractive for biomedical applications [160]. Based on Sanchez’s observations, it is known that the amount of PAA covering the magnetic core affects the size and polydispersity of nanoparticles: the greater the proportion of PAA, the smaller and more monodisperse the nanoparticles that were obtained [161].

Hamidrez and co-authors modified magnetic nanoparticles coated with a mixture of PAA and chitosan with two proteins: BSA (Bovine Serum Albumin) and IgG (Immunoglobulin G). The surface of the nanoparticles was first coated with chitosan in order to stabilize the structure; then, polyacrylic acid (PAA) was used as the outer layer [162]. After incubation with BSA and IgG protein, it was noted that the adsorption of BSA was very low in contrast to IgG protein binding. These results indicate that magnetic nanoparticles coated with polyacrylic acid (PAA) and chitosan mixture can be a good carrier for the transport of drugs [163].

An interesting application of magnetic nanoparticles coated with polyacrylic acid (PAA) was described by Huang et al. [164]. PAA-coated magnetite nanoparticles were obtained by co-precipitation and then used for the immobilization of the lipase from *Candida rugosa* from aqueous solutions [165]. It was shown that the maximum lipase adsorption was found to be 0.605 mg of enzyme per 1 mg of nanoparticles. Additionally, the desorption process and the enzyme activity after this process were investigated. The percentage of enzyme desorption was about 80%, while the enzyme activity recovery was 95.5% of the initial lipase activity value before the immobilization process [164].

Ma et al. used polyacrylic acid (PAA)-coated magnetic nanoparticles in the treatment of arterial embolism [166]. Tissue plasminogen activator (rtPA), an enzyme responsible for the fibrinolysis process, was immobilized on such surface-modified nanoparticles, using the amide bond formed with EDC/NHS. The system prepared by this way was used in an animal model to test its effectiveness in the physiological process of breaking down an arterial thrombus. The rtPA activity tested after immobilization was about 87% of the initial enzyme activity. Moreover, the results described in the conclusions indicate that after 75 min, it was possible to improve the patency of the artery to 82%, and the used material did not adversely affect the number of blood cells and hemoglobin [166]. 

**Figure 13:**
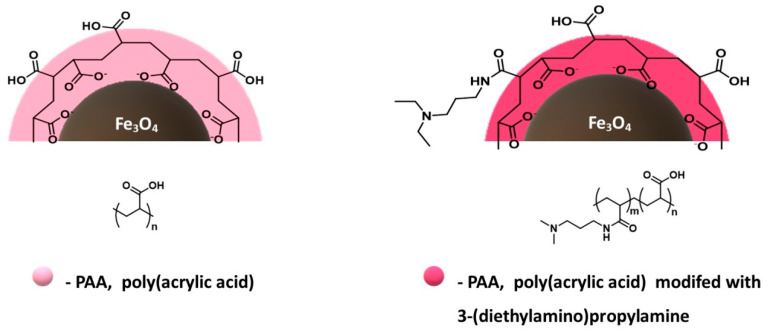
Magnetite nanoparticles coated with polyacrylic acid (PAA) and 3-(diethylamino)propylamine-modified PAA.

In the case of the design and synthesis of materials as drug carriers, it is important to study their interaction with serum proteins. Zhao and co-authors [167] studied the interactions of lysozyme (LYZ) and bovine serum albumin (BSA) with two types of magnetic nanoparticles: coated with polyacrylic acid (PAA) and the other type resulting from the modification of PAA-coated MNPs with 3-(diethylamino) propylamine (DEAPA) (Figure 13) [168]. Poly(acrylic acid) modified with (PAA)-co-3-(diethylamino)-propylamine (DEAPA)) is an important zwitterionic polymer with positively and negatively charged moieties on different monomer units.

The results of the study of the interaction of these nanoparticles with LYZ and BSA showed that the lysozyme has a high affinity for nanoparticles coated with pure, unmodified PAA, in contrast to BSA, which deposited small amounts on this material. For nanoparticles coated with PAA modified with 3-(diethylamino) propylamine, it was observed that both proteins LYZ and BSA were adsorbed in significant amounts, which was higher than for nanoparticles coated with PAA alone [167].

#### 5.1.6. Immobilization of Proteins on Nanoparticles Coated with Poly(methacrylic acid) (PMAA)

Poly(methacrylic acid) (PMAA) is a polymer synthesized from methacrylic acid, which is a viscous liquid with a specific smell. PMAA has a pKa of ≈4.8, which means that at neutral pH, the methacrylic acid groups in the lattice are almost completely deprotonated, making it an anionic polymer. PMAA can act as a polyelectrolyte with the ability to absorb and hold water. It was shown that PMAA used for a magnetic nanoparticles surface coating prevents nanoparticles aggregation and agglomeration [169,170,171]. 

Mexeriwattana and co-authors [172] investigated the interactions of blood serum proteins with poly (methacrylic acid) (PMAA)-coated magnetite nanoparticles obtained by the co-precipitation method [173]. PMAA-coated and bare magnetite nanoparticles were subjected to interaction with fetal calf serum (FCS). It has been shown that modification of the surface with poly (methacrylic acid) reduces the degree of coverage of the material by blood serum proteins compared to pure magnetite nanoparticles [172].

#### 5.1.7. Immobilization of Proteins on Nanoparticles Coated with poly(*N*-isopropylacrylamide) (PNIPAM)

Poly(*N*-isopropylacrylamide) is a temperature-sensitive polymer. It is widely studied due to its water solubility and lower critical solution temperature (LCST) close to the physiological temperature value (about 36.5–37.5 °C) [174]. Poly(*N*-isopropylacrylamide) has a relatively simple structure based on a hydrophobic skeleton and strongly hydrophilic amide groups (-CONH_2_) substituted with isopropyl moiety [175]. When heated in water above 32 °C, it undergoes a reversible phase transition with a lower critical solution temperature (LCST) from the swollen hydrated to a contracted dehydrated state, losing about 90% of its volume. It was notice that magnetic nanoparticles coated with poly(*N*-isopropylacrylamide) (PNIPAM) are characterized by thermal resistance and high magnetization as well as antibacterial properties [174,176].

**Figure 14:**
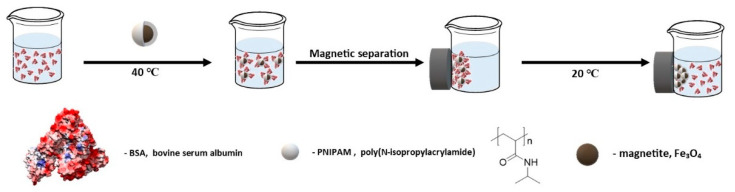
Adsorption and desorption scheme of protein on thermosensitive-polymer-coated magnetic particles.

Shamim et al. published a study on the effects of temperature, pH, and ionic strength on the adsorption and desorption of bovine serum albumin (BSA) on magnetic nanoparticles coated with a PNIPAM layer [177]. The influence of the incubation temperature on the amount of bound protein was also investigated. It was found that the amount of adsorbed protein was greater at higher temperatures (Figure 14). This behavior is attributed to the hydrophobic and hydrophilic properties of the nanomagnetic particles above and below the PNIPAM LCST, respectively. The effect of pH was also investigated, and it was observed that less protein was adsorbed at higher pH value. It was probably due to the electrostatic repulsion force between the protein molecules and the polymer shell covering the nanoparticles. The maximum amount of protein was adsorbed near the isoelectric point of BSA. The process of BSA desorption from the surface of nanoparticles was also investigated. The results showed that more protein was desorbed when adsorption was performed at the lower temperatures with the yield exceeded 80%.

An equally interesting potential application of poly(*N*-isopropylacrylamide) (PNIPAM)-coated magnetic nanoparticles has been described by Dionigi et al. [178]. The material obtained by co-precipitation was used as a matrix for the immobilization of vascular endothelial growth factor (VEGF). This article explores the possibility of delivering a cell growth factor, such as VEGF, under cell-friendly conditions to assure a high level of cell viability of HUVEC, (Primary Human Umbilical Vein Endothelial Cells) a well-known class of human cells. PNIPAM-coated magnetite nanoparticles incubated with HUVEC and loaded with VEGF demonstrated the release of the latter at 37–38 °C. The effect of the release of VEGF on the proliferation of cultivated HUVEC demonstrated both the loading and the preservation of the biological characteristics of the released VEGF. No adverse effect on cell proliferation was detected from the presence of MNPs. Summarizing, the PNIPAM-coated MNPs can be therefore considered as a promising material for controlled release of VEGF or other proteins able to stimulate vascular cells inside a scaffold.

### 5.2. Immobilization of Proteins on Nanoparticles Coated with Natural Polymers

Polymers of natural origin, the so-called biopolymers, are macromolecules especially used in biomedical sciences, including for the preparation of biocompatible and non-toxic materials. Due to the presence of reactive functional groups in their structure, these macromolecules can be easily subjected to chemical modification toward materials with better performance properties than the original biopolymer. 

#### 5.2.1. Immobilization of Proteins on Nanoparticles Coated with Chitosan (CS)

Chitosan (CS) is one of the most widely used biopolymers for magnetite core stabilization. This material is natural polysaccharide containing varying amounts of statistically decomposed structural units of 2-acetamido-2-deoxy-*β*-D-glucopyranose, (*N*-acetylglucosamine) and 2-amino-2-deoxy-*β*-D-glucopyranose (‘-glucosamine) connected via *β*—(1→4)—glycosidic bonds [179]. Chitosan has proven antiviral, anti-inflammatory, analgesic, and antibacterial activity [180,181]. It is non-toxic, biocompatible, and biodegradable polymer. It was proven that coating magnetic nanoparticles with CS does not change the thermal and magnetic properties of the magnetite material. One-pot synthesis in the presence of low molecular weight CS showed that it can protect nanoparticles from aggregation due to electrostatic repulsion between positively charged nanoparticles. Chitosan-coated nanoparticles can easily penetrate cell membranes, which is often used in biomedical research. Free hydroxyl and amine groups of chitosan allow the surface modification of nanoparticles [182,183,184,185]. In an acidic environment, free CS amino groups gain a positive charge, thanks to which they can react with negatively charged groups of nucleic acids, which are used, among others, in MRI imaging [186]. Park et al. in the review on chitosan describe its numerous applications in the delivery of low molecular weight drugs [187]. 

**Figure 15:**
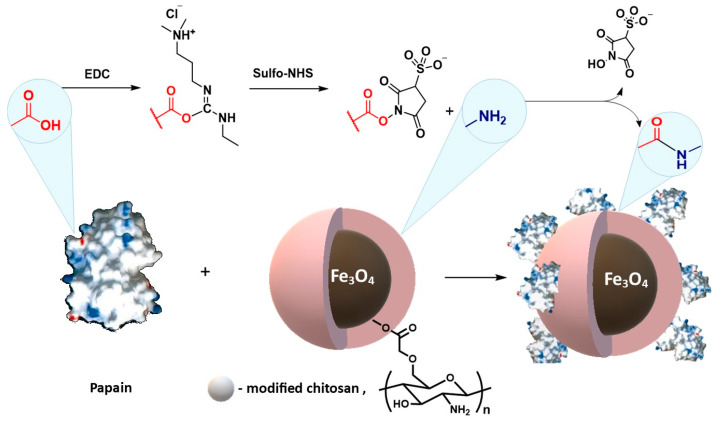
Schematic illustration for the surface modification of Fe_3_O_4_ magnetic nanoparticles by carboxymethylated chitosan and subsequent conjugation with papain.

There are many literature reports on the synthesis of chitosan-coated magnetic nanoparticles for the immobilization of catalytic proteins. Liang and Zhang [188] carried out the immobilization of papain from *Carica papaya* on magnetic nanoparticles coated with carboxymethyl chitosan. Magnetite nanoparticles were obtained by co-precipitation, and chitosan, before coating, was modified with monochloroacetic acid. Next, the chemical immobilization was performed and 1-ethyl-3-(3-dimethylaminopropyl) carbodiimide (EDC)/N-hydroxysulfosuccinimide (Sulfo-NHS) was used to couple the enzyme with the polymer (Figure 15). Conjugated papain showed increased enzymatic activity, better tolerance to pH and temperature changes, and increased storage stability compared to the native enzyme form. 

In 2008, Li and co-authors synthesized chitosan-coated magnetic nanoparticles for the immobilization of *Saccharomyces cerevisiae* almond dehydrogenase (SCMD) [189]. Dehydrogenase was physically immobilized on the MNPs surface, and the effect of adsorption on its activity was examined. For this purpose, an SCMD-catalyzed reduction reaction of phenylglyoxylic acid to (R)-mandelic acid was used. After determination of the enzyme activity in the free and immobilized form at the temperature of 25 ° C and pH 7.0, it was shown that after immobilization, the enzyme retained about 50% of its activity compared to the free form of the protein. The possibility of immobilized SCMD reuse was also investigated. After each cycle, the immobilized SCMD was recovered by magnetic separation and recycled for the reduction of phenylglyoxylic acid. The activity of the first batch was taken as 100%. After seven catalytic cycles, the remaining activity was about 48.26% of the first use. 

Wang [190] and Li and co-authors [189] obtained magnetic nanoparticles coated with chitosan cross-linked with glutaraldehyde and used them as a support for *Alcalase 2.4L* alkaline protease. The immobilization of *Alcalase 2.4L* alkaline protease on chitosan-coated magnetic nanoparticles caused an increase in enzyme activity, and the optimal range of temperature and pH profile was also significantly extended. As a model reaction to check enzyme activity, the hydrolysis of soy protein isolate (SPI) was performed by free and immobilized enzyme. The test results showed that the degree of SPI hydrolysis after 140 min was 18.38% for the immobilized enzyme and 17.50% for the free enzyme form. In addition, immobilized alkaline protease *Alcalase 2.4L* maintained approximately 86% of its original activity after ten cycles of reuse.

In turn, Sojitra et al. used chitosan-coated MNPS for pectinase immobilization [191]. Enzyme was chemically bonded to the MNPs surface with dextran polyaldehyde as a linker. Immobilization parameters such as linker concentration, time of immobilization, and support to enzyme ratio were optimized. Studies have shown that the thermal stability of pectinase immobilized on the surface of nanoparticles is twice as high as that of the free enzyme in the 55–75 °C temperature range. Moreover, the activity of immobilized pectinase was about 85% after seven reuse cycles and retained up to 89% after fifteen days of storage. Next, the obtained biocatalytic system was used for apple juice clarification. During this process, the turbidity was reduced to 74% after 150 min of juice treatment. In addition to obtaining a catalytic system that preserves the enzyme activity, this work showed that dextran polyaldehyde is a good linker for the chemical immobilization of enzymes on the surface of chitosan-coated nanoparticles. This immobilization technique includes a renewable and biocompatible natural biopolymer as a functionalizing and linking agent, making it an environmentally friendly technique and safer for workers compared to traditional chemicals. 

Articles describing the immobilization of lipases on magnetic nanoparticles coated with chitosan have been published by Kuo, Wang, Monteiro, Hosseini, Ziegler-Borowska, Sikora, and Siodmiak [182,183,192,193,194,195,196,197]. 

Kuo et al. [195] used the response surface methodology (RSM) to find the optimal lipase immobilization conditions and to investigate the factors affecting the activity of the immobilized enzyme. It was shown that the optimal immobilization conditions were 2.14 h immobilization time, pH 6.37, and enzyme/carrier ratio 0.73 (*w*/*w*). The highest lipase activity was 20 U/g of chitosan-coated nanoparticles. Additionally, after twenty repeated cycles, the immobilized lipase retained over 83% of its initial activity. The immobilized enzyme showed better operational stability, including wider thermal and pH ranges than native protein, and it remains stable after 13 days of storage at 25 °C.

**Figure 16:**
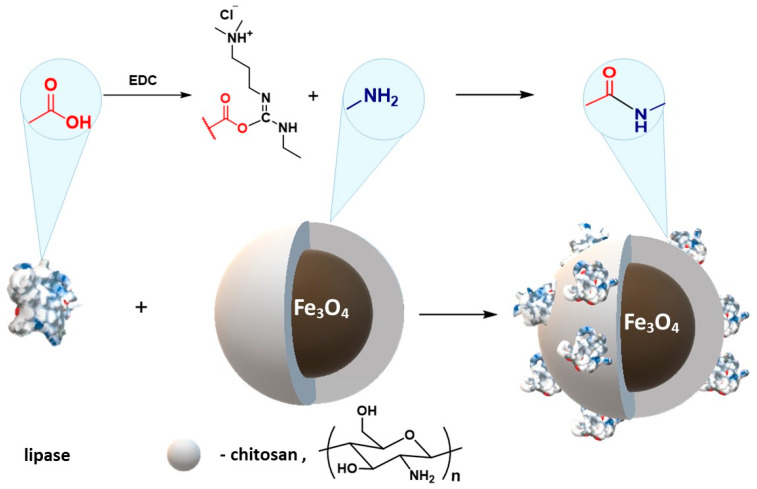
Scheme of lipase immobilization on the magnetic nanoparticles (MNPs) surface using 1-ethyl-3-(3-dimethyl-aminopropyl) carbodiimide (EDC)/sulfo-*N*-hydroxysuccinimide (NHS).

Wang et al. [196] describe the immobilization of lipase from *Thermomyces lanuginosus*. Chitosan-coated magnetic nanoparticles were prepared by a simple in situ co-precipitation that was used to covalently immobilize the enzyme via chemical conjugation after electrostatic entrapment (CCEE) (Figure 16).

As optimal immobilization conditions, the protein/carrier ratio 19.8 mg/g, pH 5.0, time 4 h, and temperature 30 °C were used. A high immobilization efficiency at the level of 75% and a bounded protein amount of 16.8 mg/g of the carrier were obtained. Moreover, the immobilized lipase retained about 70% of its initial activity after ten catalytic cycles. The prepared catalytic system was used for the synthesis of ascorbyl palmitate, which resulted with above 50% conversion of ascorbic acid to the appropriate ester. Based on these data, it was noticed that the immobilization of lipase on magnetic nanoparticles coated with chitosan by the CCEE method is an efficient and simple way to obtain a stable catalytic system.

Monteiro et al. [197] investigated the immobilization of lipase A from *Candida antarctica*. Chitosan-coated magnetite nanoparticles were activated with glutaraldehyde, and Lipase A from *Candida antarctica* was attached to the carrier surface by covalent bonding with 84% immobilization efficiency. Additionally, the immobilized biocatalyst showed a half-life about 8–11 times longer than for the free enzyme at solution with pH 5–9 and greater activity at almost all tested pH values.

**Figure 17:**
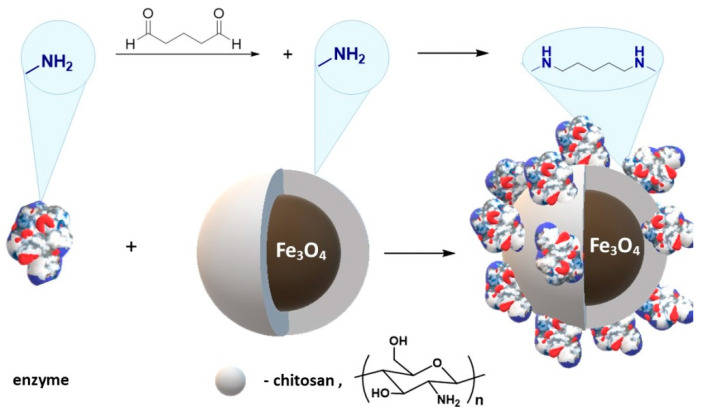
Enzyme immobilization on chitosan-coated magnetite nanoparticles with glutaraldehyde as a linker.

Hosseini et al. [194] and Monteiro [197] synthesized chitosan coated magnetic nanoparticles in two steps. In the first step, FeCl_3_·6H_2_O and sodium acetate reacted in ethylene glycol, and then the polymer shell was crosslinked with citric acid with the use hydroxyl and amine groups of chitosan at neutral pH. The resulting nanoparticles were used for Lipase B from *Candida antarctica* immobilization with glutaraldehyde as the linker (Figure 17). The results showed that the immobilized enzyme has higher storage stability than free protein. It has also been successfully used to itaconic anhydride oligomerization by ring-opening esterification. This process is a green approach for the production of functional oligoesters and can be applied to make photo-curable esters.

**Figure 18:**
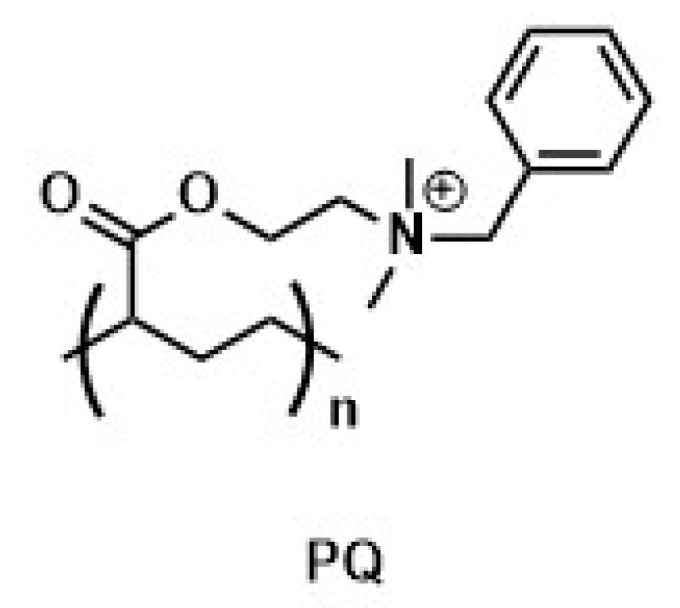
Structure of poly [N-benzyl-2-(methacryloxy)-N, N-dimethylethanaminium bromide] (PQ).

Ziegler-Borowska et al. [182] synthesized magnetite nanoparticles with surface modified with a mixture of two polymers: chitosan and poly [N-benzyl-2-(methacryloxy)-N, N-dimethylethanaminium bromide] (PQ) (Figure 18).

Nanoparticles with different mass ratios of these polymers were used for the immobilization of lipase from *Candida rugosa*. The enzyme was covalently bounded to the nanoparticle surface by EDC/sulfo-NHS activation. The activity recovery of the immobilized enzyme was estimated by the hydrolysis of olive oil, and its maximum value was about 82% for CS–PQ nanoparticles (1:1). The residual activity of immobilized lipase was over 90% after five catalytic cycles and remained at the level of 70–72.1% after 10 cycles. Moreover, it was noticed that the presence of the quaternary ammonium salt had a positive effect on the dispersion and stability of the solutions due to the spatial effects and electrostatic repulsion of polymer chains. As a result of quaternary ammonium salt biological activity, the studied magnetic nanoparticles may be also of particular importance in antimicrobial applications. On the other hand, Siódmiak and co-workers used this catalytic system for kinetic separation through enantioselective esterification of (R, S)-ibuprofen—a widely used non-steroidal anti-inflammatory drug [183]. Lipase, immobilized on the surface of CS/PQ magnetic carriers with (EDC)/(sulfo-NHS) activation showed high catalytic activity, which allowed obtaining (S)-methyl ibuprofen ester with high enantioselectivity (E = 50.6). Moreover, the properties of this magnetite particles allow for better optimization of the enantioselective esterification of (R, S)-ibuprofen and as a result could reduce the overall cost of this reaction. In addition, the use of this nanocatalytic system allowed for maintaining high enantioselective activity after repeated use.

**Figure 19:**
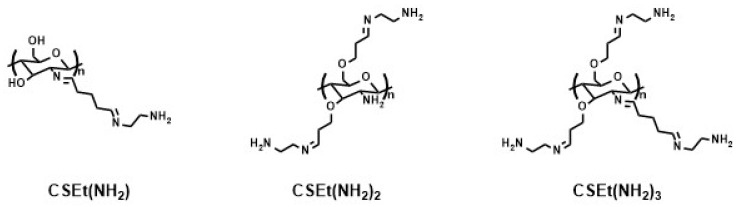
Structure of the aminated chitosan Fe_3_O_4_-CSEt(NH_2_)_1–3_.

Later, Ziegler-Borowska and co-authors prepared magnetite nanoparticles coated with chemically-modified chitosan rich in free amino groups remote from the polymer chain (Figure 19) [183,185,198,199,200,201,202]. First, chitosan-coated magnetite nanoparticles were synthesized by an in situ co-precipitation reaction in an alkaline solution. Then, the reactive chitosan groups, hydroxyl and amino, were used for its functionalization, obtaining three polymer coatings with different content of amino groups able to bind protein (Figure 19). Prepared nanoparticles were used for the chemical immobilization of proteins: lipase from *Candida rugosa* [183,193,201], human serum albumin (HSA) [185,199], and the androgen receptor (AR) [202]. 

Sikora and co-authors [193,201] used these magnetic nanoparticles with immobilized lipase from *Candida rugosa* for the enantioselective acetylation of (R, S)-atenolol. Additionally, the catalytic activity of two types of commercially available lipases from *Candida rugosa* immobilized on two different magnetic nanoparticles was compared. Among all the tested catalytic systems, the best results were obtained with the lipase from *Candida rugosa* immobilized on Fe_3_O_4_-CS-Et(NH_2_)_1_ (E = 66.9, c = 41.84%, ee = 94.1%). Additionally, these studies also showed that even after five catalytic cycles, the immobilized lipase maintains high catalytic activity. Next, Marszałł et al. [202] used these same supports for immobilization of the androgen receptor (AR). The research involved comparing various AR carriers, such as silica-coated magnetic nanoparticles and chitosan-coated nanoparticles with varying amounts of amino groups. Immobilization was performed in two ways: by covalent immobilization of the AR via an amino terminal group or available carboxyl groups. The initial characterization of AR-coated magnetic nanoparticles was performed with dihydrotestosterone as a well-known AR ligand. Subsequently, chitosan-modified nanoparticles with distant primary amine groups (Fe_3_O_4_-CS-Et(NH_2_)_3_) were used for the isolation of AR ligands (bicalutamide, flutamide, hydroxyflutamide, and levonogestrel) from the mixture. Based on the obtained results, it was noticed that the selected nanoparticles are a promising semi-quantitative tool for the identification of compounds with high affinity for AR and may be of particular importance in the identification of new agonists or antiandrogens. Finally, these materials were used for HSA immobilization. Moreover, the influence of the selected method of functionalization of the polymer coating covering magnetite nanoparticles (MNP) as well as drying methods of the carrier on the immobilization of human serum albumin HSA was performed [185]. Albumin was immobilized on three types of nanoparticles coated with aminated chitosan with different content of amino groups at a large distance from the Fe_3_O_4_-CS-Et(NH_2_)_1–3_ surface. It was noticed that both the synthesis method and the method of drying nanoparticles have a large impact on the effectiveness of immobilization. The largest amount of protein was immobilized on Fe_3_O_4_-CS-Et (NH_2_)_3_, and it was 210.32 mg/g nanoparticles. In the case of the materials Fe_3_O_4_-CSEt(NH_2_) and Fe_3_O_4_-CS-Et(NH_2_)_3_ dried by the freeze-drying method, the efficiency of protein immobilization was 200% higher than in the case of nanoparticles dried in a vacuum dryer. In another work, Ziegler-Borowska et al. used such immobilized albumin for protein–drug interaction study [184].

The synthesis of magnetite nanoparticles coated with a mixture of chitosan and collagen with the use of various cross-linking agents of the polymer coating, glutaraldehyde (Glu) and squaric acid (SqA), was also described by Ziegler-Borowska and co-authors [192]. Obtained nanomaterials were prepared in a standard co-precipitation reaction and then coated with chitosan (CS), collagen (Coll), and a mixture of these two biopolymers and cross-linked. As a result of the performed syntheses, six types of nanomaterials were obtained: CS/Glu (Fe_3_O_4_), Coll/Glu (Fe_3_O_4_), CS–Coll/Glu (Fe_3_O_4_), Coll/SqA(Fe_3_O_4_), CS/SqA (Fe_3_O_4_), and CS–Coll/SqA(Fe_3_O_4_). Subsequently, lipase from *Candida rugosa* immobilization was carried out on the surface of the obtained nanoparticles with EDC/NHS activation. The best results in terms of recovered lipase activity and specific activities were observed for nanoparticles with a polymer coating cross-linked with squaric acid. The specific activity of lipase immobilized on SqA cross-linked materials was 52 U/mg lipase, and it was about two times higher than for the enzyme immobilized on nanomaterial with glutaraldehyde 26 U/mg lipase. Additionally, after the fifth catalytic cycle, the residual activity for all tested magnetic nanoparticles was about 80–90%, and at the end of the tenth cycle, the immobilized lipases still retained almost 80% of their activity. Moreover, a little hyperactivation of lipase immobilized on CS/SqA (Fe_3_O_4_) and CS–Coll/SqA(Fe_3_O_4_) nanoparticles was observed.

#### 5.2.2. Immobilization of Proteins on Nanoparticles Coated with Agarose

**Figure 20:**
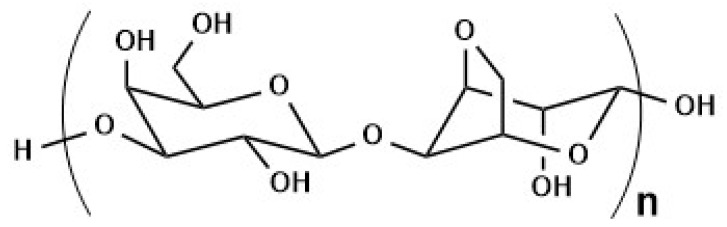
The structure of agarose.

Agarose is a polysaccharide usually obtained from some red seaweed. It is a linear polymer composed of the agarobiose repeating unit, which is a disaccharide consisting of D-galactose that is well soluble in water 3,6-anhydro-L-galactopyranose (Figure 20) [203,204]. 

After cooling of its aqueous solution, this inert polymer forms thermo-reversible gels. The biocompatibility of agarose hydrogels and the mild conditions required for their gelling make the composite hydrogel widely used in the biomedical industry. As a result of the non-toxicity of agarose-coated MNPs, they are widely used in drug delivery systems [205,206,207].

Chen and co-authors synthesized agarose-coated magnetic nanoparticles for *β*-glucosidase (BGL) immobilization [208]. Magnetite nanoparticles coupled with agarose (AMNPs) were prepared via co-precipitation reaction in alkaline condition with span–80 surfactants addition in organic solvent. Next, iminodiacetate was attached to the MNPs through epichlorohydrin agent and then chelated with metal ions. Enzyme immobilization was performed by the physical adsorption. Cobalt ion chelated nanoparticles prepared by this way showed a high enzyme adsorption capacity of 1.81 mg/g nanoparticles. Moreover, a hyperactivation of immobilized *β*-glucosidase was observed (enzyme activity of 117% per gram of protein in *β*-glucosidase immobilization). Additionally, compared to free BGL, the immobilized enzyme showed higher hydrolytic activity and thermostability and better operational stability. After running for 15 catalytic cycles, this catalytic system retained over 90% of its initial activity. 

#### 5.2.3. Immobilization of Proteins on Nanoparticles Coated with Starch

**Figure 21:**
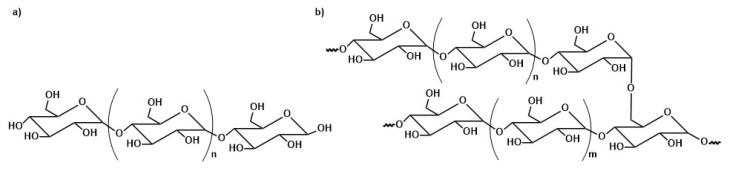
Chemical structure of starch components: (**a**) amylopectin and (**b**) amylose.

Starch is one of the most interesting natural polysaccharides used for MNPs coating. It is a plant polysaccharide, consisting only of glucose units linked by α-glycosidic bonds, acting as an energy store in plants [209]. Starch hydrolyses only to α-D-glucose but is not a chemically homogeneous compound—it consists of two fractions: unbranched amylose and branched amylopectin (Figure 21). 

The presence of highly reactive hydroxyl groups and the relatively good solubility of starch make it susceptible to modifications, which inspires the creation of new materials with potential properties such as biodegradability and non-toxicity [210]. For chemical modifications of this polysaccharide oxidation, esterification, and etherification reactions are used [211,212]. It was also confirmed that starch-coated magnetite nanoparticles retain their magnetic properties [213] and are characterized with high biocompatibility as well as colloidal stability. As a result of that, starch and modified starch-coated magnetite nanoparticles can be considered as one of the biomaterials with biological potential for applications in the biomedical department [214,215]. 

**Figure 22:**
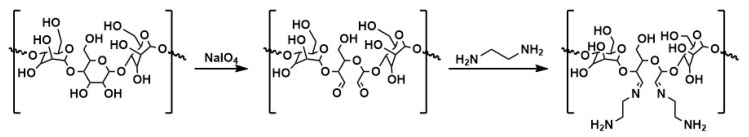
Synthesis of aminated starch.

Ziegler-Borowska [212] carried out the synthesis of starch and modified starch-coated magnetite nanoparticles. First, starch-coated MNPs were obtained by in situ co-precipitation, and the polymer shell was modified in the next stage: it was oxidized to dialdehyde starch and then simply and quickly aminated without solvent (pounding in a mortar) to nanoparticles coated with starch enriched with amino groups (Figure 22).

These magnetite nanoparticles were used as a carrier for HSA immobilization with glutaraldehyde as the binding agent. The amount of HSA immobilized on the nanoparticles was about 149.96 mg HSA/g of the carrier, and the protein retained its activity after immobilization. Next, these HSA-aminated starch-coated MNPs were used for HSA-ketoprofen binding study [185].

Wang et al. used starch-coated magnetic nanoparticles for pectinase produced by Penicillium oxalicum F67 (PoPase) immobilization [216]. Before the immobilization process, magnetic nanoparticles coated with corn starch were cross-linked with glutaraldehyde. Enzyme immobilization on the support was performed by physical adsorption. Additionally, the influence of various factors on the rate of enzyme regeneration after immobilization was investigated. Next, free and immobilized enzymes were used to extract apple juice to evaluate their effect on juice yield. The studies showed that after eight catalytic cycles, the immobilized enzymes retained about 60% of the initial PoPase activity. This confirmed that starch-coated magnetic nanoparticles are a good carrier for PoPase immobilization, which may be applicable to juice processing.

Gagnon and co-authors [217] described the use of starch-coated magnetic nanoparticles for the capture of monoclonal IgG antibodies. A hybrid system that embodies elements of both PEG precipitation and steric exclusion chromatography on starch-coated 200 nm nanoparticles enables the magnetic capture of 78 mg IgG per mg of particles, with 98% antibody recovery.

Wensheng Lu and co-authors published the synthesis of magnetic nanoparticles coated with dialdehyde starch [218]. They oxidized starch with sodium periodate; then, the nanoparticles were cross-linked with epichlorohydrin solution, and bovine serum albumin (BSA) was successfully immobilized on the carrier surface. The support prepared in this way can be used for targeted drug release.

In 2008, Kuroiwa and co-authors described the immobilization of the enzyme chitosanase on magnetic nanoparticles coated with amylose [219]. As previously mentioned, amylose is a polysaccharide that is a constituent of starch. Enzyme immobilization on the support was carried out by two different techniques—physical adsorption and multiple covalent bonding at different concentrations of the immobilized enzyme. The yield of the chemical immobilization was higher than that of physical adsorption. A high protein immobilization capacity (maximum 0.4 g protein/g molecule) was observed for these nanoparticles. Next, chitosanase immobilized on magnetic amylose-coated nanoparticles was used to produce chitosan oligosaccharides with high physiological activity. The physiologically active pentamers and hexamers of chitosan oligosaccharide were obtained by this way in high yield (40%), which was higher than for conventional methods. 

#### 5.2.4. Immobilization of Proteins on Nanoparticles Coated with Cellulose

Cellulose is an unbranched biopolymer composed of D-glucose units linked linearly with *β*-1,4-glycosidic bonds [220]. Cellulose is the main structural component of plants and can also be produced by marine animals, algae, fungi, bacteria, invertebrates, and the pulpit, which make it the most abundant polymer in nature [221,222]. Coating nanoparticles with cellulose results in the formation of a hydrophilic surface that prevents agglomeration, increases the dispersion of nanoparticles in physiological solution, and increases bioavailability; it is also a suitable coating for functionalization [223].

Aguilera and co-authors showed that magnetic nanoparticles coated with carboxymethyl cellulose—a cellulose derivative—have great potential as drug delivery systems during neurological treatment [223]. The biodegradability of the coating allows the drug to be delivered at a controlled and steady rate to the target site. Moreover, such materials are able to cross the blood–brain barrier (BBB) [223]. 

**Figure 23:**
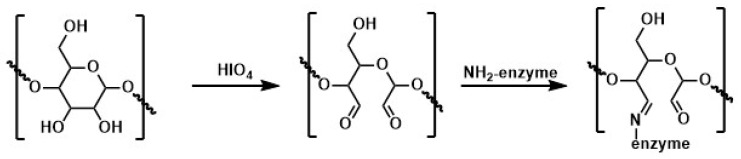
Scheme of dialdehyde cellulose synthesis and enzyme immobilization.

Namdeo and co-authors described the synthesis of magnetic nanoparticles coated with cellulose for the immobilization of α-amylase [224]. Before immobilization, the cellulose coating was subjected to chemical modifications-oxidation with periodic acid (Figure 23). 

Amylase was covalently immobilized on this support, and it was noticed that nanoparticles coated with 16 and 28 wt % of cellulose bounded about 9.1 and 16.2 mg enzyme/g nanoparticles, respectively. Therefore, it has been proven that immobilized α-amylose retains its ability for starch degradation. Later, Ivanova and co-authors also published the results of immobilization of α-amylase on magnetic nanoparticles coated with cellulose [225]. Another application of these magnetic nanoparticles was presented by Anirudhan and co-authors [226]. Magnetic nanoparticles coated with cellulose were used for heme protein adsorption. Nanoparticles were obtained by co-precipitation with nanocrystalline cellulose, which was further modified by free radical graft copolymerization using VSA (vinylsulfonic acid) and MAA (methacrylic acid). It has been found that this material can be an effective myoglobin adsorbent from aqueous solutions. The adsorption was affected by parameters such as time, temperature, concentration, and pH. The best recovery of myoglobin was achieved at pH 7.0, near the isoelectric point of the protein, indicating the existence of electrostatic interaction between the adsorbent and the protein. 

Guo and co-authors used magnetic nanoparticles coated with modified cellulose for protein separation [227]. First, primary hydroxyl groups on the cellulose surface were selectively oxidized with TEMPO (2,2,6,6-tetramethylpiperidinyloxy). At the same time, magnetite nanoparticles functionalized with amino groups were synthesized by co-precipitation reaction and coupled to previously modified cellulose via EDC/sulfoNHS linking. In the next step, MNPs were complexed with copper ions to provide specific binding sites for proteins. The prepared material showed high lysozyme binding capacity (860.6 ± 14.6 mg/g) as well as efficient reutilization for protein separation. Total protein recovery using simple elution was approximately 98%.

Lately, Mohammadi and co-authors focused on the interaction of bovine α-lactalbumin (BLA) with variously coated magnetic nanoparticles: naked, dopamine stabilized, and coated with cellulose [228]. They showed that magnetic nanoparticles coated with cellulose have the highest protein binding affinity compared to the rest. Additionally, the circular dichroism spectra revealed that the BLA conformation was preserved after interaction with magnetic nanoparticles. This important feature, in addition to the exceptionally strong binding affinity, makes these particles novel model nanostructures for nanomedicine-focused applications.

#### 5.2.5. Immobilization of Proteins on Nanoparticles Coated with Dextran

Dextran is another natural polysaccharide-based polymer applied for magnetite nanoparticles coating [229,230,231]. It is a water-soluble macromolecule consisting of many glucose molecules linked by α-1,6-glycosidic bonds. It was shown that magnetic nanoparticles coating with hydrophilic organic polymers such as dextran improve their drug delivery properties and colloidal stability [232,233,234]. Moreover, hydroxyl groups of dextran structure can be modified and functionalized with primary amines, which affects their ability to bind targeted ligands [235]. 

Weissleder and al. showed that magnetic nanoparticles coated with transferrin (Tf) could be used to image the transferrin receptor by magnetic resonance imaging (MRI) [236]. These results can be related to the expression of this receptor. After the administration of magnetic nanoparticles conjugated with transferrin to the body, their high accumulation in cancer cells was observed, which allows their use as a probe for imaging neoplastic sites by MRI [236]. Despite high doses of the probe, the observed signal in MRI imaging showed very low values. Therefore, in the same year, Hogemann et al. describe the synthesis of novel nanoparticles for magnetic resonance imaging (MRI) of transferrin receptors characterized by a much better signal [237]. These nanoparticles were based on magnetite core coated with dextran and immobilized transferrin. The best imaging results were obtained for MNPs coated with cross-linked dextran (CL10) and immobilized oxidized transferrin (Tf-SH) with N-succinimidyl 3-(2-pyridyldithio) propionate (SPDP) as a linker. It was showed that the use of this linker does not affect the activity of immobilized transferrin (Tf). It was also proved that in such a system, four transferrin (Tf) molecules were immobilized on one nanoparticle surface. This make it possible to reduce the amount of potential dose injected into the body in order to obtain the appropriate intensity of imaging [237]. 

An equally interesting use of magnetic nanoparticles coated with dextran was presented by Horng et al., which used this system for the anti-C-reactive protein (CRP) antibody immobilization [238]. Prepared magnetic nanoparticles were applied to the C-reactive protein (CRP) detection with use a SQUID (superconducting quantum interference device) gradiometer. It is well known that C-reactive protein (CRP) is produced by the body during tissue damage, necrosis, and ongoing inflammation, and it is a non-specific marker of these phenomena. Standard for CRP detection, commercially available ELISA [239] assays are used. Results obtained for dextran-coated MPNs showed CRP detection at about 10 ng/mL. This value, compared to standard procedures using ELISA tests, is lower by an order of magnitude, which significantly improves the measurement possibilities [238]. 

**Figure 24:**
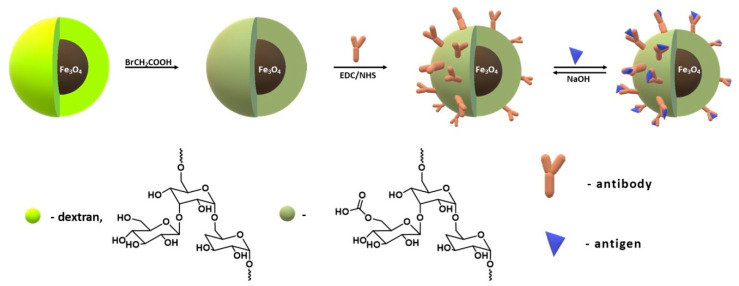
Synthesis of carboxymethylated dextran-coated magnetic nanoparticles (CMD-MNPs) covered with anti-bovine serum albumin (BSA) antibody and subsequent capture and release of BSA in sample solution.

The immobilization of the antibody on carboxymethylated dextran (CMD)-coated magnetic nanoparticles was presented by Li et al. (Figure 24) [240]. Anti-BSA was immobilized on the nanoparticles surface with EDC/NHS activation to couple the amino groups of the antibody and CMC carboxyl groups (Figure 24). Next, this system was tested for the ability to trap BSA.

The results obtained in this study indicate that over 20 mg of BSA was bounded per 1 g of anti-BSA immobilized on CMD-coated nanoparticles. Moreover, the potential use of such systems for selective proteins capture was mentioned [240].

Carboxymethylated dextran (CMD) was also used by Vasić and co-authors in their research for alcohol dehydrogenase (ADH) immobilization [234]. It was shown that the remaining ADH activity after immobilization at 4 °C oscillates around only 25%.

Ziv and co-authors [241] describe the body’s reactions measured by the amount of antibodies produced against magnetic nanoparticles coated with dextran and gelatin with immobilized HSA (human serum albumin) (Gel-MNP-Dex-HSA). The synthesis of such a system was performed with the innovative method of nucleation. This procedure consists of a controlled growth of magnetic nanoparticles coated with gelatin from a gelatin solution to which iron salts II and III have been added. In the final stage, HSA was immobilized using the previously prepared coating and the Michael addition reaction. The body’s response to these systems was tested by measuring the number of produced antibodies (anti-gelatin, anti-dextran, and anti-HSA). In conclusion, the authors indicate the correctness of using the gelatin nucleation method due to the very weak immunogenic nature of this polymer. However, regarding dextran, it was found that it could be replaced with another biopolymer of lower immunogenicity. Investigating the immunogenicity of such materials is extremely important from the point of view of biomedical applications; therefore, Ziv et al. plan further studies of nanomaterials in this area [241]. 

## 6. Conclusions

Summarizing the collected information, it can be stated that every year, the use of magnetite nanoparticles coated with polymers for protein immobilization is increasing. The design, synthesis, and application of nanoparticles coated with biopolymers in their original form and after chemical modifications are developing particularly dynamically. Among the proteins, enzymes are still most often immobilized on magnetite particles, which is most likely due to the possibility of their reuse in the catalytic cycle. Nevertheless, there are more and more works on the immobilization of serum proteins and antibodies. Table 4 below summarizes all the most important applications of magnetite nanoparticles in protein immobilization discussed in the above work. Such a summary, divided into the polymer covering the magnetite core and the immobilized protein, can be of convenience for the reader.

## Figures and Tables

**Table 1:** Chemical methods for the synthesis of magnetic nanoparticles; their advantages and disadvantages [19,20,64].

Methods	Advantages	Disadvantages
Chemical Co-precipitation	simple and effective	not suitable for the preparation of high-purity accurate stoichiometric phase
Thermal Decomposition	particle size and shapes are controllable	time-consuming synthesis at high temperatures
Microemulsion	uniform properties	surfactants are difficult to remove; small amount can be synthesized
Hydrothermal	particle size and shapes are easily controllable homogeneity	high pressure and high temperature reaction
Sonochemical	size distribution in narrow particle	mechanism not still understood
Electrochemical	easy to control particle size	reproducibility

**Table 2:** Advantages and disadvantages of most commonly known immobilization methods [114,118,119].

Method of Immobilization	Binding Nature	Advantages	Disadvantages
Adsorption	weak interactions such as hydrogen bond, hydrophobic, and van der Waals interactions	- does not or little affects the enzyme structure - simple, cheap, and easy - no conformational change of the protein - no need to use reagents	- low stability - non-specific adsorption

**Table 3:** Results of LC-MS/MS analysis checking which of the FBS proteins remain on the surface of bare MNPs and MNPs functionalized with polymethacrylic acid, linear polyethylenimine, and branched polyethylenimine after MNPs have been contacted with biological solution containing 10% FBS [155].

Protein	Bare MNPs	MNPs Coated with Polymethacrylic Acid	MNPs Coated with Linear Polyethylenimine	MNPs Coated with Branched Polyethylenimine
Albumin	+	+	+	+
Antithrombin	-	-	-	+
α-2-HS-glycoprotein	+	-	-	-
Inter- α-inhibitor	-	-	+	+
Apolipoprotein A-1	-	-	+	+
Apolipoprotein E	-	-	+	+
Complement Component 4A	-	-	+	+
Tetranectin	+	+	-	-
α-fetoprotein	-	-	-	+
α-1-antiproteinase	-	-	-	+
Kininogen	-	+	-	-
Complement factor H	+	-	-	-
Hemoglobin	+	+	+	+
Immunoglobulin	-	+	-	-
Complement factor I	+	-	-	-
Complement factor B	+	+	-	-
Apolipoprotein B	+	-	-	-
Lactoferrin	-	+	-	-

**Table 4:** List of proteins immobilized on magnetic nanoparticles coated with polymers described in the literature.

Protein	Polimer	References
Fetal bovine serum (FBS)	polyethylene glycol (PEG)	[125]
Cytochrome C	polyethylene glycol (PEG)	[124]
Transferrin	polyvinyl alcohol (PVA)	[131]
Horseradish peroxidase	polyvinyl alcohol/chitosan (PVA/CS)	[132]
Trypsin	polyvinyl alcohol (PVA)	[134]
Herceptin antibody (HER)	poly(D,L-lactide-co-glycolide) (PLGA)/polyvinyl alcohol (PVA)	[143]
Bovine serum albumin (BSA)	poly(D,L-lactide-co-glycolide)(PLGA)	[145]
Laccase from *Trametes versicolor*	polyethyleneimine (PEI)	[149]
Green fluorescent protein	polyethyleneimine (PEI)	[154]
Fetal bovine serum (FBS)	polyethyleneimine (PEI)	[155]
*Candida rugosa* lipase i *Mucor miehei* lipase	polyethyleneimine (PEI)	[156]
Fetal bovine serum (FBS)	polyethyleneimine (PEI)	[158]
Fetal bovine serum (FBS)	polyacrylic acid (PAA)	[158]
Fetal bovine serum (FBS)	polyacrylic acid (PAA)	[155]
Bovine serum albumin (BSA) and Immunoglobulin G	polyacrylic acid (PAA)/chitosan (CS)	[163]
Lipase from Candida Rugosa	polyacrylic acid (PAA)	[164]
Tissue plasminogen activator (rtPA)	polyacrylic acid (PAA)	[166]
Lysozyme (LYZ) and bovine serum albumin (BSA)	polyacrylic acid (PAA)	[167]
Fetal bovine serum (FBS)	polymethacrylic acid (PMAA)	[172]
Bovine serum albumin (BSA)	poly(*N*-isopropylacrylamide) (PNIPAM)	[177]
Vascular endothelial growth factor (VEGF)	poly(*N*-isopropylacrylamide) (PNIPAM)	[178]
Streptavidin	poly(*N*-isopropylacrylamide) (PNIPAM)	[242]
Papain from Carica papaya	Chitosan (CS)	[188]
*Saccharomyces cerevisiae* Mandelated dehydrogenase (SCMD)	Chitosan (CS)	[189]
Pectinase	Chitosan (CS)	[191]
Lipase from Candida rugosa	Chitosan (CS)	[195]
Lipase from *Thermomyces lanuginosus*	Chitosan (CS)	[196]
Lipase A from Candida antarctica	Chitosan (CS)	[197]
Lipase B from Candida antarctica	Chitosan (CS)	[194]
Lipase from Candida rugosa	Chitosan (CS)	[182]
Lipase from Candida rugosa	Chitosan (CS)	[183]
Lipase from Candida rugosa and Human serum albumin (HSA)	Chitosan (CS)	[198]
Lipase from Candida rugosa	Chitosan (CS)	[193]
Lipase from Candida rugosa	Chitosan (CS)	[201]
Lipase from Candida rugosa	Chitosan (CS)	[192]
Androgen receptor (AR)	Chitosan (CS)	[202]
Human serum albumin (HSA)	Chitosan (CS)	[184]
Human serum albumin (HSA)	Chitosan (CS)	[185]
β-Glucosidase (BGL)	Agarose	[208]
Human serum albumin (HSA)	Starch	[212]
Pectinase	Starch	[216]
Immunoglobulin G	Starch	[217]
Bovine serum albumin (BSA)	Starch	[218]
Chitosanase	Amylose	[219]
α-amylase	Cellulose	[224]
α-amylase	Cellulose	[225]
Heme	Cellulose	[226]
Lysozyme	Cellulose	[227]
Bovine α-lactalbumin (BLA)	Cellulose	[228]
Transferrin	Dextran	[237]
Anti-CRP antibodies	Dextran	[238]
Anti-BSA	Dextran	[240]
Alcohol dehydrogenase (ADH)	Dextran	[234]
Human serum albumin (HSA)	Dextran	[241]

## References

[1] Jeevanandam J., Barhoum A., Chan Y.S., Dufresne A., Danquah M.K. (2018). Review on nanoparticles and nanostructured materials: History, sources, toxicity and regulations. Beilstein J. Nanotechnol..

[2] Aseri A., Garg S.K., Nayak A., Trivedi S.K., Ahsan J. (2015). Magnetic nanoparticles: Magnetic nano-technology using biomedical applications and future prospects. Int. J. Pharm. Sci. Rev. Res..

[3] Dzhardimalieva G.I., Pomogailo A.D., Rozenberg A.S., Leonowicz M. (2009). Magnetic Metallopolymer Nanocomposites: Preparation and Properties. Magn. Nanoparticles.

[4] Kolesnichenko V.L. (2009). Synthesis of Nanoparticulate Magnetic Materials.

[5] Vedmedenko E. (2007). Competing Interactions and Patterns in Nanoworld. The Chemistry of Nanomaterials Nanoparticles Introduction to Nanotechnology.

[6] Can M.M., Coşkun M., Firat T. (2012). A comparative study of nanosized iron oxide particles: Magnetite (Fe_3_O_4_), maghemite (γ-Fe_2_O_3_) and hematite (α-Fe_2_O_3_), using ferromagnetic resonance. J. Alloys Compd..

[7] Mahdavi M., Ahmad M.B., Haron M.J., Namvar F., Nadi B., Ab Rahman M.Z., Amin J. (2013). Synthesis, surface modification and characterisation of biocompatible magnetic iron oxide nanoparticles for biomedical applications. Molecules.

[8] Zhang Z., Kong J. (2011). Novel magnetic Fe_3_O_4_@C nanoparticles as adsorbents for removal of organic dyes from aqueous solution. J. Hazard. Mater..

[9] Ito A., Shinkai M., Honda H., Kobayashi T. (2005). Medical application of functionalized magnetic nanoparticles. J. Biosci. Bioeng..

[10] Garcia-Galan C., Berenguer-Murcia Á., Fernandez-Lafuente R., Rodrigues R.C. (2011). Potential of different enzyme immobilization strategies to improve enzyme performance. Adv. Synth. Catal..

[11] Liu X., Zhang L., Zeng J., Gao Y., Tang Z. (2013). Superparamagnetic nano-immunobeads toward food safety insurance. J. Nanoparticle Res..

[12] Rahim S., Iftikhar F.J., Malik M.I. (2020). Biomedical Applications of Magnetic Nanoparticles.

[13] Magro M., Venerando A., Macone A., Canettieri G., Agostinelli E., Vianello F. (2020). Nanotechnology-based strategies to develop new anticancer therapies. Biomolecules.

[14] Long N.V., Yang Y., Teranishi T., Thi C.M., Cao Y., Nogami M. (2015). Biomedical applications of advanced multifunctional magnetic nanoparticles. J. Nanosci. Nanotechnol..

[15] Banerjee R., Katsenovich Y., Lagos L., McIintosh M., Zhang X., Li C.-Z. (2010). Nanomedicine: Magnetic Nanoparticles and their Biomedical Applications. Curr. Med. Chem..

[16] Wallyn J., Anton N., Vandamme T.F. (2019). Synthesis, principles, and properties of magnetite nanoparticles for in vivo imaging applications—A review. Pharmaceutics.

[17] Rossi L.M., Costa N.J.S., Silva F.P., Wojcieszak R. (2014). Magnetic nanomaterials in catalysis: Advanced catalysts for magnetic separation and beyond. Green Chem..

[18] Wu W., He Q., Jiang C. (2008). Magnetic iron oxide nanoparticles: Synthesis and surface functionalization strategies. Nanoscale Res. Lett..

[19] Ali A., Zafar H., Zia M., ul Haq I., Phull A.R., Ali J.S., Hussain A. (2016). Synthesis, characterization, applications, and challenges of iron oxide nanoparticles. Nanotechnol. Sci. Appl..

[20] Xu J., Sun J., Wang Y., Sheng J., Wang F., Sun M. (2014). Application of iron magnetic nanoparticles in protein immobilization. Molecules.

[21] Shabatina T.I., Vernaya O.I., Shabatin V.P., Melnikov M.Y. (2020). Magnetic Nanoparticles for Biomedical Purposes: Modern Trends and Prospects. Magnetochemistry.

[22] Rehse K. (1989). Kurzmi tteilung Vergleichende Untersuchungen zur Bindung nichtsteroidaler Antirheumatika an Humanserumalbumin und deren Interaktion rnit Phenprocoumon. Archiv der Pharmazie.

[23] Holm J., Babol L.N., Markova N., Lawaetz A.J., Hansen S.I. (2014). The interrelationship between ligand binding and thermal unfolding of the folate binding protein. the role of self-association and pH. Biochim. Biophys. Acta Proteins Proteom..

[24] Wahab R.A., Elias N., Abdullah F., Ghoshal S.K. (2020). On the taught new tricks of enzymes immobilization: An all-inclusive overview. React. Funct. Polym..

[25] Rehm F.B.H., Chen S., Rehm B.H.A. (2016). Enzyme engineering for in situ immobilization. Molecules.

[26] Hoarau M., Badieyan S., Marsh E.N.G. (2017). Immobilized enzymes: Understanding enzyme-surface interactions at the molecular level. Org. Biomol. Chem..

[27] Leitgeb M., Knez Ž., Vasić K. (2016). Micro-and Nanocarriers for Immobilization of Enzymes. Micro and Nanotechnologies for Biotechnology.

[28] Philippova O., Barabanova A., Molchanov V., Khokhlov A. (2011). Magnetic polymer beads: Recent trends and developments in synthetic design and applications. Eur. Polym. J..

[29] Khan I., Saeed K., Khan I. (2019). Nanoparticles: Properties, applications and toxicities. Arab. J. Chem..

[30] Yurkov G.Y., Gubin S.P., Ovchenkov E.A. (2009). Magnetic Nanocomposites Based on the Metal-Containing (Fe, Co, Ni) Nanoparticles inside the Polyethylene Matrix. Magn. Nanoparticles.

[31] Hanemann T., Szabó D.V. (2010). Polymer-Nanoparticle Composites: From Synthesis to Modern Applications. Materials.

[32] Alnoch R.C., Dos Santos L.A., de Almeida J.M., Krieger N., Mateo C. (2020). Recent trends in biomaterials for immobilization of lipases for application in non-conventional media. Catalysts.

[33] Bezerra C.S., De Farias Lemos C.M.G., De Sousa M., Gonçalves L.R.B. (2015). Enzyme immobilization onto renewable polymeric matrixes: Past, present, and future trends. J. Appl. Polym. Sci..

[34] Zdarta J., Meyer A.S., Jesionowski T., Pinelo M. (2018). A general overview of support materials for enzyme immobilization: Characteristics, properties, practical utility. Catalysts.

[35] Khan U.S., Khattak N.S., Rahman A., Khan F. (2011). Historical development of magnetite nanoparticles synthesis. J. Chem. Soc. Pakistan.

[36] Benelmekki M., Benelmekki M. (2014). An introduction to nanoparticles and nanotechnology. Des. Hybrid Nanoparticles.

[37] Sugimoto T., Matijević E. (1980). Formation of uniform spherical magnetite particles by crystallization from ferrous hydroxide gels. J. Colloid Interface Sci..

[38] Sun S., Zeng H. (2002). Size-controlled synthesis of magnetite nanoparticles. J. Am. Chem. Soc..

[39] Hasany S.F., Ahmed I., Rajan J., Rehman A. (2013). Systematic Review of the Preparation Techniques of Iron Oxide Magnetic Nanoparticles. Nanosci. Nanotechnol..

[40] Jiang W., Lai K.L., Hu H., Zeng X.B., Lan F., Liu K.X., Wu Y., Gu Z.W. (2011). The effect of [Fe^3+^]/[Fe^2+^] molar ratio and iron salts concentration on the properties of superparamagnetic iron oxide nanoparticles in the water/ethanol/toluene system. J. Nanoparticle Res..

[41] Khurshid H., Li W., Chandra S., Phan M.H., Hadjipanayis G.C., Mukherjee P., Srikanth H. (2013). Mechanism and controlled growth of shape and size variant core/shell FeO/Fe_3_O_4_ nanoparticles. Nanoscale.

[42] Fried T., Shemer G., Markovich G. (2001). Ordered two-dimensional arrays of ferrite nanoparticles. Adv. Mater..

[43] Kassabova-Zhetcheva V.D., Pavlova L.P., Samuneva B.I., Cherkezova-Zheleva Z.P., Mitov I.G., Mikhov M.T. (2007). Characterization of superparamagnetic MgxZn1-x Fe_2_O_4_ powders. Cent. Eur. J. Chem..

[44] Nemati Z., Alonso J., Martinez L.M., Khurshid H., Garaio E., Garcia J.A., Phan M.H., Srikanth H. (2016). Enhanced Magnetic Hyperthermia in Iron Oxide Nano-Octopods: Size and Anisotropy Effects. J. Phys. Chem. C.

[45] Alonso J., Barandiarán J.M., Fernández Barquín L., García-Arribas A., El-Gendy J.M., Barandiarán J.M., Hadimani R.L. (2018). Magnetic Nanoparticles, Synthesis, Properties, and Applications.

[46] Solans C., Izquierdo P., Nolla J., Azemar N., Garcia-Celma M.J. (2005). Nano-emulsions. Curr. Opin. Colloid Interface Sci..

[47] Pileni M.P. (1993). Reverse micelles as microreactors. J. Phys. Chem..

[48] Chin A.B., Yaacob I.I. (2007). Synthesis and characterization of magnetic iron oxide nanoparticles via w/o microemulsion and Massart’s procedure. J. Mater. Process. Technol..

[49] Biehl P., von der Lühe M., Dutz S., Schacher F.H. (2018). Synthesis, characterization, and applications of magnetic nanoparticles featuring polyzwitterionic coatings. Polymers.

[50] Baker I. (2018). Magnetic Nanoparticle Synthesis.

[51] Tang B., Yuan L., Shi T., Yu L., Zhu Y. (2009). Preparation of nano-sized magnetic particles from spent pickling liquors by ultrasonic-assisted chemical co-precipitation. J. Hazard. Mater..

[52] Wang Y., Nkurikiyimfura I., Pan Z. (2015). Sonochemical Synthesis of Magnetic Nanoparticles. Chem. Eng. Commun..

[53] Mathew D.S., Juang R.S. (2007). An overview of the structure and magnetism of spinel ferrite nanoparticles and their synthesis in microemulsions. Chem. Eng. J..

[54] Bang J.H., Suslick K.S. (2007). Sonochemical Synthesis of Nanosized Hollow Hematite. J. Am. Chem. Soc..

[55] Vijayakumar R., Koltypin Y., Felner I., Gedanken A. (2000). Sonochemical synthesis and characterization of pure nanometer-sized Fe_3_O_4_ particles. Mater. Sci. Eng. A.

[56] Weng Y.C., Rusakova I.A., Baikalov A., Chen J.W., Wu N.L. (2005). Microstructural evolution of nanocrystalline magnetite synthesized by electrocoagulation. J. Mater. Res..

[57] Cabrera L., Gutierrez S., Menendez N., Morales M.P., Herrasti P. (2008). Magnetite nanoparticles: Electrochemical synthesis and characterization. Electrochim. Acta.

[58] Ibrahim M., Serrano K.G., Noe L., Garcia C., Verelst M. (2009). Electro-precipitation of magnetite nanoparticles: An electrochemical study. Electrochim. Acta.

[59] Franger S., Berthet P., Berthon J. (2004). Electrochemical synthesis of Fe_3_O_4_ nanoparticles in alkaline aqueous solutions containing complexing agents. J. Solid State Electrochem..

[60] Mosivand S., Monzon L.M.A., Ackland K., Kazeminezhad I., Coey J.M.D. (2013). The effect of organics on the structure and magnetization of electro-synthesised magnetite nanoparticles. J. Nanoparticle Res..

[61] Fajaroh F., Setyawan H., Sutrisno, Nazriati, Wonorahardjo S. (2014). To enhance the purity and crystallinity of magnetite nanoparticles prepared by surfactant-free electrochemical method by imposing higher voltage. AIP Conf. Proc..

[62] Marques R.F.C., Garcia C., Lecante P., Ribeiro S.J.L., Noé L., Silva N.J.O., Amaral V.S., Millán A., Verelst M. (2008). Electro-precipitation of Fe_3_O_4_ nanoparticles in ethanol. J. Magn. Magn. Mater..

[63] Marín T., Ortega D., Montoya P., Arnache O., Calderón J. (2014). A new contribution to the study of the electrosynthesis of magnetic nanoparticles: The influence of the supporting electrolyte. J. Appl. Electrochem..

[64] Xu J.K., Zhang F.F., Sun J.J., Sheng J., Wang F., Sun M. (2014). Bio and nanomaterials based on Fe_3_O_4_. Molecules.

[65] Xia T., Wang J., Wu C., Meng F., Shi Z., Lian J., Feng J., Meng J. (2012). Novel complex-coprecipitation route to form high quality triethanolamine-coated Fe_3_O_4_ nanocrystals: Their high saturation magnetizations and excellent water treatment properties. CrystEngComm.

[66] Muthiah M., Park I.K., Cho C.S. (2013). Surface modification of iron oxide nanoparticles by biocompatible polymers for tissue imaging and targeting. Biotechnol. Adv..

[67] Gautam A., Van Veggel F.C.J.M. (2013). Synthesis of nanoparticles, their biocompatibility, and toxicity behavior for biomedical applications. J. Mater. Chem. B.

[68] Kydralieva K.A., Dzhardimalieva G.I., Yurishcheva A.A., Jorobekova S.J. (2016). Nanoparticles of Magnetite in Polymer Matrices: Synthesis and Properties. J. Inorg. Organomet. Polym. Mater..

[69] Arias L.S., Pessan J.P., Vieira A.P.M., De Lima T.M.T., Delbem A.C.B., Monteiro D.R. (2018). Iron oxide nanoparticles for biomedical applications: A perspective on synthesis, drugs, antimicrobial activity, and toxicity. Antibiotics.

[70] Sahoo Y., Pizem H., Fried T., Golodnitsky D., Burstein L., Sukenik C.N., Markovich G., Gan R. (2001). Alkyl Phosphonate/Phosphate Coating on Magnetite Nanoparticles: A Comparison with Fatty Acids. Langmuir.

[71] Wang Y., Wong J.F., Teng X., Lin X.Z., Yang H. (2003). “Pulling” Nanoparticles into Water: Phase Transfer of Oleic Acid Stabilized Monodisperse Nanoparticles into Aqueous Solutions of α-Cyclodextrin. Nano Lett..

[72] Luchini A., Heenan R.K., Paduano L., Vitiello G. (2016). Functionalized SPIONs: The surfactant nature modulates the self-assembly and cluster formation. Phys. Chem. Chem. Phys..

[73] Wu W., Wu Z., Yu T., Jiang C., Kim W.S. (2015). Recent progress on magnetic iron oxide nanoparticles: Synthesis, surface functional strategies and biomedical applications. Sci. Technol. Adv. Mater..

[74] Zhu N., Ji H., Yu P., Niu J., Farooq M.U., Akram M.W., Udego I.O., Li H., Niu X. (2018). Surface modification of magnetic iron oxide nanoparticles. Nanomaterials.

[75] Wang J., Zhou H., Zhuang J., Liu Q. (2015). Magnetic γ-Fe_2_O_3_, Fe_3_O_4_, and Fe nanoparticles confined within ordered mesoporous carbons as efficient microwave absorbers. Phys. Chem. Chem. Phys..

[76] Liu X., Ma Y., Zhang Q., Zheng Z., Wang L.-S., Peng D.L. (2018). Facile synthesis of Fe_3_O_4_/C composites for broadband microwave absorption properties. Appl. Surf. Sci..

[77] Lu A.H., Salabas E.L., Schüth F. (2007). Magnetic nanoparticles: Synthesis, protection, functionalization, and application. Angew. Chemie Int. Ed..

[78] Gupta A.K., Gupta M. (2005). Synthesis and surface engineering of iron oxide nanoparticles for biomedical applications. Biomaterials.

[79] Wagner J., Autenrieth T., Hempelmann R. (2002). Core shell particles consisting of cobalt ferrite and silica as model ferrofluids [CoFe_2_O_4_-SiO_2_ core shell particles]. J. Magn. Magn. Mater..

[80] Couto D., Freitas M., Carvalho F., Fernandes E. (2015). Iron Oxide Nanoparticles: An Insight into their Biomedical Applications. Curr. Med. Chem..

[81] Costa C., Brandão F., Bessa M.J., Costa S., Valdiglesias V., Kiliç G., Fernández-Bertólez N., Quaresma P., Pereira E., Pásaro E. (2016). In vitro cytotoxicity of superparamagnetic iron oxide nanoparticles on neuronal and glial cells. Evaluation of nanoparticle interference with viability tests. J. Appl. Toxicol..

[82] Raghunath A., Perumal E. (2017). Metal oxide nanoparticles as antimicrobial agents: A promise for the future. Int. J. Antimicrob. Agents.

[83] Thorek D.L.J., Tsourkas A. (2008). Size, charge and concentration dependent uptake of iron oxide particles by non-phagocytic cells. Biomaterials.

[84] Zhu X.M., Wang Y.X.J., Leung K.C.F., Lee S.F., Zhao F., Wang D.W., Lai J.M.Y., Wan C., Cheng C.H.K., Ahuja A.T. (2012). Enhanced cellular uptake of aminosilane-coated superparamagnetic iron oxide nanoparticles in mammalian cell lines. Int. J. Nanomed..

[85] Ling W., Wang M., Xiong C., Xie D., Chen Q., Chu X., Qiu X., Li Y., Xiao X. (2019). Synthesis, surface modification, and applications of magnetic iron oxide nanoparticles. J. Mater. Res..

[86] Lee M.H., Leu C.C., Lin C.C., Tseng Y.F., Lin H.Y., Yang C.N. (2019). Gold-decorated magnetic nanoparticles modified with hairpin-shaped DNA for fluorometric discrimination of single-base mismatch DNA. Microchim. Acta.

[87] Wu W., Jiang C., Roy V.A.L. (2015). Recent progress in magnetic iron oxide-semiconductor composite nanomaterials as promising photocatalysts. Nanoscale.

[88] Wu W., Xiao X., Zhang S., Ren F., Jiang C. (2011). Facile method to synthesize magnetic iron oxides/TiO_2_ hybrid nanoparticles and their photodegradation application of methylene blue. Nanoscale Res. Lett..

[89] Xu H., Ouyang S., Liu L., Reunchan P., Umezawa N., Ye J. (2014). Recent advances in TiO2-based photocatalysis. J. Mater. Chem. A.

[90] Wu W., Zhang S., Ren F., Xiao X., Zhou J., Jiang C. (2011). Controlled synthesis of magnetic iron oxides@SnO 2 quasi-hollow core-shell heterostructures: Formation mechanism, and enhanced photocatalytic activity. Nanoscale.

[91] Li S.K., Huang F.Z., Wang Y., Shen Y.H., Qiu L.G., Xie A.J., Xu S.J. (2011). Magnetic Fe_3_O_4_@C@Cu_2_O composites with bean-like core/shell nanostructures: Synthesis, properties and application in recyclable photocatalytic degradation of dye pollutants. J. Mater. Chem..

[92] Saffari J., Mir N., Ghanbari D., Khandan-Barani K., Hassanabadi A., Hosseini-Tabatabaei M.R. (2015). Sonochemical synthesis of Fe_3_O_4_/ZnO magnetic nanocomposites and their application in photo-catalytic degradation of various organic dyes. J. Mater. Sci. Mater. Electron..

[93] Joseph J., Nishad K.K., Sharma M., Gupta D.K., Singh R.R., Pandey R.K. (2012). Fe_3_O_4_ and CdS based bifunctional core-shell nanostructure. Mater. Res. Bull..

[94] Liu L., Xiao L., Zhu H.Y., Shi X.W. (2013). Studies on interaction and illumination damage of CS-Fe_3_O_4_@ZnS:Mn to bovine serum albumin. J. Nanoparticle Res..

[95] Zhou W., Chen Y., Wang X., Gu Z., Hu Y. (2011). Synthesis of Fe_3_O_4_@PbS hybrid nanoparticles through the combination of surface-initiated atom transfer radical polymerization and acidolysis by H 2S. J. Nanosci. Nanotechnol..

[96] Luo S., Chai F., Zhang L., Wang C., Li L., Liu X., Su Z. (2012). Facile and fast synthesis of urchin-shaped Fe_3_O_4_@Bi_2_S_3_ core-shell hierarchical structures and their magnetically recyclable photocatalytic activity. J. Mater. Chem..

[97] Rădulescu M., Andronescu E., Holban A.M., Vasile B.S., Iordache F., Mogoantă L., Dan Mogoșanu G., Grumezescu A.M., Georgescu M., Chifiriuc M.C. (2016). Antimicrobial nanostructured bioactive coating based on Fe_3_O_4_ and patchouli oil for wound dressing. Metals.

[98] Mu Q., Kievit F.M., Kant R.J., Lin G., Jeon M., Zhang M. (2015). Anti-HER2/neu peptide-conjugated iron oxide nanoparticles for targeted delivery of paclitaxel to breast cancer cells. Nanoscale.

[99] Jahanban-Esfahlan A., Dastmalchi S., Davaran S. (2016). A simple improved desolvation method for the rapid preparation of albumin nanoparticles. Int. J. Biol. Macromol..

[100] Nosrati H., Sefidi N., Sharafi A., Danafar H., Manjili H.K. (2018). Bovine Serum Albumin (BSA) coated iron oxide magnetic nanoparticles as biocompatible carriers for curcumin-anticancer drug. Bioorg. Chem..

[101] McBain S.C., Yiu H.H.P., Dobson J. (2008). Magnetic nanoparticles for gene and drug delivery. Int. J. Nanomed..

[102] Kharisov B.I., Eldin M.S.M. (2018). Enzyme Immobilization: Nanopolymers for Enzyme Immobilization Applications. CRC Concise Encycl. Nanotechnol..

[103] Nelson J.M., Griffin E.G. (1916). Adsorption of invertase. J. Am. Chem. Soc..

[104] Mohamad N.R., Marzuki N.H.C., Buang N.A., Huyop F., Wahab R.A. (2015). An overview of technologies for immobilization of enzymes and surface analysis techniques for immobilized enzymes. Biotechnol. Biotechnol. Equip..

[105] Cao L. (2006). Carrier-bound Immobilized Enzymes: Principles, Application and Design. Carrier-bound Immobil. Enzym. Princ. Appl. Des..

[106] Homaei A.A., Sariri R., Vianello F., Stevanato R. (2013). Enzyme immobilization: An update. J. Chem. Biol..

[107] Ahmad R., Sardar M. (2015). Enzyme Immobilization: An Overview on Nanoparticles as Immobilization Matrix. Biochem. Anal. Biochem..

[108] Polshettiwar V., Luque R., Fihri A., Zhu H., Bouhrara M., Basset J.M. (2011). Magnetically recoverable nanocatalysts. Chem. Rev..

[109] Nisha S., Karthick S.A., Gobi N. (2012). A Review on Methods, Application and Properties of Immobilized Enzyme. Chem. Sci. Rev. Lett..

[110] Rao S.V., Anderson K.W., Bachas L.G. (1998). Oriented Immobilization of Proteins. Mikrochim. Acta.

[111] Lee C.H., Lin T.S., Mou C.Y. (2009). Mesoporous materials for encapsulating enzymes. Nano Today.

[112] Yiitolu M., Temoçin Z. (2010). Immobilization of Candida rugosa lipase on glutaraldehyde-activated polyester fiber and its application for hydrolysis of some vegetable oils. J. Mol. Catal. B Enzym..

[113] Lai B.H., Yeh C.C., Chen D.H. (2012). Surface modification of iron oxide nanoparticles with polyarginine as a highly positively charged magnetic nano-adsorbent for fast and effective recovery of acid proteins. Process Biochem..

[114] Thangaraj B., Solomon P.R. (2019). Immobilization of Lipases–A Review. Part I: Enzyme Immobilization. ChemBioEng Rev..

[115] Wickramathilaka M.P., Tao B.Y. (2019). Characterization of covalent crosslinking strategies for synthesizing DNA-based bioconjugates. J. Biol. Eng..

[116] Vashist S.K., Zhang B., Zheng D., Al-Rubeaan K., Luong J.H.T., Sheu F.S. (2011). Sulfo-N-hydroxysuccinimide interferes with bicinchoninic acid protein assay. Anal. Biochem..

[117] Bart J., Tiggelaar R., Yang M., Schlautmann S., Zuilhof H., Gardeniers H. (2009). Room-temperature intermediate layer bonding for microfluidic devices. Lab Chip.

[118] Eş I., Vieira J.D.G., Amaral A.C. (2015). Principles, techniques, and applications of biocatalyst immobilization for industrial application. Appl. Microbiol. Biotechnol..

[119] Chakraborty S., Rusli H., Nath A., Sikder J., Bhattacharjee C., Curcio S., Drioli E. (2016). Immobilized biocatalytic process development and potential application in membrane separation: A review. Crit. Rev. Biotechnol..

[120] Bhatia S. (2016). Natural Polymer Drug Delivery Systems: Nanoparticles, Plants, and Algae.

[121] Mohiti-Asli M., Loboa E.G. (2016). Nanofibrous Smart Bandages for Wound Care.

[122] Kim J.E., Shin J.Y., Cho M.H. (2012). Magnetic nanoparticles: An update of application for drug delivery and possible toxic eVects. Arch. Toxicol..

[123] Tai M.F., Lai C.W., Abdul Hamid S.B. (2016). Facile Synthesis Polyethylene Glycol Coated Magnetite Nanoparticles for High Colloidal Stability. J. Nanomater..

[124] Mukhopadhyay A., Joshi N., Chattopadhyay K., De G. (2012). A facile synthesis of PEG-coated magnetite (Fe_3_O_4_) nanoparticles and their prevention of the reduction of cytochrome C. ACS Appl. Mater. Interfaces.

[125] Chang M., Chang Y.J., Chao P.Y., Yu Q. (2018). Exosome purification based on peg-coated Fe_3_O_4_ Nanoparticles. PLoS ONE.

[126] Hoshino A., Costa-Silva B., Shen T.L., Rodrigues G., Hashimoto A., Mark M.T., Molina H., Kohsaka S., Di Giannatale A., Ceder S. (2015). Tumour exosome integrins determine organotropic metastasis. Nature.

[127] Zhang X., Yuan X., Shi H., Wu L., Qian H., Xu W. (2015). Exosomes in cancer: Small particle, big player. J. Hematol. Oncol..

[128] Rahayu L.B.H., Wulandari I.O., Santjojo D.H., Sabarudin A. (2018). Synthesis and Characterization of Fe_3_O_4_ Nanoparticles with Polyvinyl Alcohol (PVA) as Capping Agent and Glutaraldehyde as Crosslinkers. Nature B.

[129] Hassan C.M., Peppas N.A. (2000). Structure and applications of poly(vinyl alcohol) hydrogels produced by conventional crosslinking or by freezing/thawing methods. Adv. Polym. Sci..

[130] Nadeem M., Ahmad M., Akhtar M.S., Shaari A., Riaz S., Naseem S., Masood M., Saeed M.A. (2016). Magnetic properties of polyvinyl alcohol and doxorubicine loaded iron oxide nanoparticles for anticancer drug delivery applications. PLoS ONE.

[131] Mahmoudi M., Shokrgozar M.A., Sardari S., Moghadam M.K., Vali H., Laurent S., Stroeve P. (2011). Irreversible changes in protein conformation due to interaction with superparamagnetic iron oxide nanoparticles. Nanoscale.

[132] Laochai T., Mooltongchun M., Teepoo S. (2016). Design and Construction of Magnetic Nanoparticles Incorporated with a Chitosan and Poly (vinyl) Alcohol Cryogel and its Application for Immobilization of Horseradish Peroxidase. Energy Procedia.

[133] Ali M., Tahir M.N., Siwy Z., Neumann R., Tremel W., Ensinger W. (2011). Hydrogen peroxide sensing with horseradish peroxidase-modified polymer single conical nanochannels. Anal. Chem..

[134] Sahin S., Ozmen I. (2020). Covalent immobilization of trypsin on polyvinyl alcohol-coated magnetic nanoparticles activated with glutaraldehyde. J. Pharm. Biomed. Anal..

[135] Gerlt J.A. (1999). Stabilization of Reactive Intermediates and Transition States in Enzyme Active Sites by Hydrogen Bonding. Compr. Nat. Prod. Chem..

[136] Lü J.M., Wang X., Marin-Muller C., Wang H., Lin P.H., Yao Q., Chen C. (2011). Current advances in research and clinical applications of PLGA-based nanotechnology. Expert Rev. Mol. Diagn..

[137] Hirenkumar M., Steven S. (2012). Poly Lactic-co-Glycolic Acid (PLGA) as Biodegradable Controlled Drug Delivery Carrier. Polymers.

[138] Sun X., Xu C., Wu G., Ye Q., Wang C. (2017). Review poly(lactic-co-glycolic acid): Applications and future prospects for periodontal tissue regeneration. Polymers.

[139] Danhier F., Ansorena E., Silva J.M., Coco R., Le Breton A., Préat V. (2012). PLGA-based nanoparticles: An overview of biomedical applications. J. Control. Release.

[140] Ganipineni L.P., Ucakar B., Joudiou N., Bianco J., Danhier P., Zhao M., Bastiancich C., Gallez B., Danhier F., Préat V. (2018). Magnetic targeting of paclitaxel-loaded poly(lactic-co-glycolic acid)-based nanoparticles for the treatment of glioblastoma. Int. J. Nanomed..

[141] Spillmann C.M., Naciri J., Algar W.R., Medintz I.L., Delehanty J.B. (2014). Multifunctional liquid crystal nanoparticles for intracellular fluorescent imaging and drug delivery. ACS Nano.

[142] Key J., Leary J.F. (2014). Nanoparticles for multimodal in vivo imaging in nanomedicine. Int. J. Nanomed..

[143] Yang J., Lee C.H., Park J., Seo S., Lim E.K., Song Y.J., Suh J.S., Yoon H.G., Huh Y.M., Haam S. (2007). Antibody conjugated magnetic PLGA nanoparticles for diagnosis and treatment of breast cancer. J. Mater. Chem..

[144] Thorn C.F., Oshiro C., Marsh S., Hernandez-Boussard T., McLeod H., Klein T.E., Altman R.B. (2011). Doxorubicin pathways: Pharmacodynamics and adverse effects. Pharm. Genom..

[145] Shubhra Q.T.H., Tóth J., Gyenis J., Feczkó T. (2014). Surface modification of HSA containing magnetic PLGA nanoparticles by poloxamer to decrease plasma protein adsorption. Colloids Surfaces B Biointerfaces.

[146] Lu W., Ling M., Jia M., Huang P., Li C., Yan B. (2014). Facile synthesis and characterization of polyethylenimine-coated Fe_3_O_4_ superparamagnetic nanoparticles for cancer cell separation. Mol. Med. Rep..

[147] Wang X., Zhou L., Ma Y., Li X., Gu H. (2009). Control of aggregate size of polyethyleneimine-coated magnetic nanoparticles for magnetofection. Nano Res..

[148] Kwolek U., Wójcik K., Janiczek M., Nowakowska M., Kepczynski M. (2017). Synthesis and antibacterial properties of quaternary ammonium derivative of polyethylenimine. Polimery.

[149] Xia T.T., Liu C.Z., Hu J.H., Guo C. (2016). Improved performance of immobilized laccase on amine-functioned magnetic Fe_3_O_4_ nanoparticles modified with polyethylenimine. Chem. Eng. J..

[150] Fernández-Fernández M., Sanromán M.Á., Moldes D. (2013). Recent developments and applications of immobilized laccase. Biotechnol. Adv..

[151] Crestini C., Perazzini R., Saladino R. (2010). Oxidative functionalisation of lignin by layer-by-layer immobilised laccases and laccase microcapsules. Appl. Catal. A Gen..

[152] Giardina P., Faraco V., Pezzella C., Piscitelli A., Vanhulle S., Sannia G. (2010). Laccases: A never-ending story. Cell. Mol. Life Sci..

[153] Hahn V., Meister M., Hussy S., Cordes A., Enderle G., Saningong A., Schauer F. (2018). Enhanced laccase-mediated transformation of diclofenac and flufenamic acid in the presence of bisphenol A and testing of an enzymatic membrane reactor. AMB Express.

[154] Zuvin M., Kuruoglu E., Kaya V.O., Unal O., Kutlu O., Yagci Acar H., Gozuacik D., Kosar A. (2019). Magnetofection of green fluorescent protein encoding DNA-bearing polyethyleneimine-coated superparamagnetic iron oxide nanoparticles to human breast cancer cells. ACS Omega.

[155] Wiogo H.T.R., Lim M., Bulmus V., Gutiérrez L., Woodward R.C., Amal R. (2012). Insight into serum protein interactions with functionalized magnetic nanoparticles in biological media. Langmuir.

[156] Kannan K., Mukherjee J., Gupta M.N. (2013). Use of polyethyleneimine coated Fe_3_O_4_ nanoparticles as an ion-exchanger for protein separation. Sci. Adv. Mater..

[157] Gräfe C., Weidner A., Lühe M.V.D., Bergemann C., Schacher F.H., Clement J.H., Dutz S. (2016). Intentional formation of a protein corona on nanoparticles: Serum concentration affects protein corona mass, surface charge, and nanoparticle-cell interaction. Int. J. Biochem. Cell Biol..

[158] Calatayud M.P., Sanz B., Raffa V., Riggio C., Ibarra M.R., Goya G.F. (2014). The effect of surface charge of functionalized Fe_3_O_4_ nanoparticles on protein adsorption and cell uptake. Biomaterials.

[159] Roohi F., Lohrke J., Ide A., Schütz G., Dassler K. (2012). Studying the effect of particle size and coating type on the blood kinetics of superparamagnetic iron oxide nanoparticles. Int. J. Nanomed..

[160] Xu Y., Zhuang L., Lin H., Shen H., Li J.W. (2013). Preparation and characterization of polyacrylic acid coated magnetite nanoparticles functionalized with amino acids. Thin Solid Films.

[161] Sanchez L.M., Martin D.A., Alvarez V.A., Gonzalez J.S. (2018). Polyacrylic acid-coated iron oxide magnetic nanoparticles: The polymer molecular weight influence. Colloids Surfaces A Physicochem. Eng. Asp..

[162] Shagholani H., Ghoreishi S.M., Mousazadeh M. (2015). Improvement of interaction between PVA and chitosan via magnetite nanoparticles for drug delivery application. Int. J. Biol. Macromol..

[163] Hamidreza S. (2017). Synthesis of Nanocomposition of Poly Acrylic Acid/Chitosan Coated-Magnetite Nanoparticles to Investigation of Interaction with BSA and IGG Proteins. Int. J. Nanomater. Nanotechnol. Nanomed..

[164] Huang S.H., Liao M.H., Chen D.H. (2006). Fast and efficient recovery of lipase by polyacrylic acid-coated magnetic nano-adsorbent with high activity retention. Sep. Purif. Technol..

[165] Liao M.H., Chen D.H. (2002). Preparation and characterization of a novel magnetic nano-adsorbent. J. Mater. Chem..

[166] Ma Y.H., Wu S.Y., Wu T., Chang Y.J., Hua M.Y., Chen J.P. (2009). Magnetically targeted thrombolysis with recombinant tissue plasminogen activator bound to polyacrylic acid-coated nanoparticles. Biomaterials.

[167] Zhao T., Chen K., Gu H. (2013). Investigations on the interactions of proteins with polyampholyte-coated magnetite nanoparticles. J. Phys. Chem. B.

[168] Xiao W., Gu H., Li D., Chen D., Deng X., Jiao Z., Lin J. (2012). Microwave-assisted synthesis of magnetite nanoparticles for MR blood pool contrast agents. J. Magn. Magn. Mater..

[169] Woźniak E., Špírková M., Šlouf M., Garamus V.M., Šafaříková M., Šafařík I., Štěpánek M. (2017). Stabilization of aqueous dispersions of poly(methacrylic acid)-coated iron oxide nanoparticles by double hydrophilic block polyelectrolyte poly(ethylene oxide)-block-poly(N-methyl-2-vinylpyridinium iodide). Colloids Surfaces A Physicochem. Eng. Asp..

[170] Ahmad H., Ahmad A., Islam S.S. (2017). Magnetic Fe_3_O_4_@poly(methacrylic acid) particles for selective preconcentration of trace arsenic species. Microchim. Acta.

[171] Li K., Chen K., Wang Q., Zhang Y., Gan W. (2019). Synthesis of poly(acrylic acid) coated magnetic nanospheres via a multiple polymerization route. R. Soc. Open Sci..

[172] Mekseriwattana W., Srisuk S., Kriangsaksri R., Niamsiri N., Prapainop K. (2019). The Impact of Serum Proteins and Surface Chemistry on Magnetic Nanoparticle Colloidal Stability and Cellular Uptake in Breast Cancer Cells. AAPS PharmSciTech.

[173] Yu S., Chow C.M. (2004). Carboxyl group (-CO2H) functionalized ferrimagnetic iron oxide nanoparticles for potential bio-applications. J. Mater. Chem..

[174] Lanzalaco S., Armelin E. (2017). Poly(*N*-isopropylacrylamide) and Copolymers: A Review on Recent Progresses in Biomedical Applications. Gels.

[175] Alipour A., Shekardasht M.B., Gharbani P. (2020). The synthesis, characterization and applications of poly[:*N*-isopropylacrylamide-co-3-allyloxy-1,2-propanediol] grafted onto modified magnetic nanoparticles. RSC Adv..

[176] Nguyen N.H.A., Darwish M.S.A., Stibor I., Kejzlar P., Ševců A. (2017). Magnetic Poly(*N*-isopropylacrylamide) Nanocomposites: Effect of Preparation Method on Antibacterial Properties. Nanoscale Res. Lett..

[177] Shamim N., Hong L., Hidajat K., Uddin M.S. (2006). Thermosensitive-polymer-coated magnetic nanoparticles: Adsorption and desorption of Bovine Serum Albumin. J. Colloid Interface Sci..

[178] Dionigi C., Lungaro L., Goranov V., Riminucci A., Piñeiro-Redondo Y., Bañobre-López M., Rivas J., Dediu V. (2014). Smart magnetic poly(*N*-isopropylacrylamide) to control the release of bio-active molecules. J. Mater. Sci. Mater. Med..

[179] Cheung R.C.F., Ng T.B., Wong J.H., Chan W.Y. (2015). Chitosan: An Update on Potential Biomedical and Pharmaceutical Applications. Mar Drugs..

[180] Chirkov S.N. (2002). The antiviral activity of chitosan (review). Appl. Biochem. Microbiol..

[181] Croisier F., Jérôme C. (2013). Chitosan-based biomaterials for tissue engineering. Eur. Polym. J..

[182] Ziegler-Borowska M., Siódmiak T., Chełminiak D., Cyganiuk A., Marszałł M.P. (2014). Magnetic nanoparticles with surfaces modified with chitosan-poly[*N*-benzyl-2-(methacryloxy)-N,N-dimethylethanaminium bromide] for lipase immobilization. Appl. Surf. Sci..

[183] Siódmiak T., Ziegler-Borowska M., Marszałł M.P. (2013). Lipase-immobilized magnetic chitosan nanoparticles for kinetic resolution of (R,S)-ibuprofen. J. Mol. Catal. B Enzym..

[184] Ziegler-Borowska M., Mylkie K., Kozlowska M., Nowak P., Chelminiak-Dudkiewicz D., Kozakiewicz A., Ilnicka A., Kaczmarek-Kedziera A. (2019). Effect of geometrical structure, drying, and synthetic method on aminated chitosan-coated magnetic nanoparticles utility for HSA effective immobilization. Molecules.

[185] Ziegler-Borowska M., Mylkie K., Nowak P., Rybczynski P., Sikora A., Chelminiak-Dudkiewicz D., Kaczmarek-Kedziera A. (2020). Testing for ketoprofen binding to HSA coated magnetic nanoparticles under normal conditions and after oxidative stress. Molecules.

[186] Qiao T., Wu Y., Jin J., Gao W., Xie Q., Wang S., Zhang Y., Deng H. (2011). Conjugation of catecholamines on magnetic nanoparticles coated with sulfonated chitosan. Colloids Surfaces A Physicochem. Eng. Asp..

[187] Park J.H., Saravanakumar G., Kim K., Kwon I.C. (2010). Targeted delivery of low molecular drugs using chitosan and its derivatives. Adv. Drug Deliv. Rev..

[188] Liang Y.Y., Zhang L.M. (2007). Bioconjugation of papain on superparamagnetic nanoparticles decorated with carboxymethylated chitosan. Biomacromolecules.

[189] Li G.Y., Jiang Y.R., Huang K.L., Ding P., Yao L.L. (2008). Kinetics of adsorption of Saccharomyces cerevisiae mandelated dehydrogenase on magnetic Fe_3_O_4_-chitosan nanoparticles. Colloids Surfaces A Physicochem. Eng. Asp..

[190] Wang S.N., Zhang C.R., Qi B.K., Sui X.N., Jiang L.Z., Li Y., Wang Z.J., Feng H.X., Wang R., Zhang Q.Z. (2014). Immobilized alcalase alkaline protease on the magnetic chitosan nanoparticles used for soy protein isolate hydrolysis. Eur. Food Res. Technol..

[191] Sojitra U.V., Nadar S.S., Rathod V.K. (2017). Immobilization of pectinase onto chitosan magnetic nanoparticles by macromolecular cross-linker. Carbohydr. Polym..

[192] Ziegler-Borowska M., Chelminiak-Dudkiewicz D., Siódmiak T., Sikora A., Wegrzynowska-Drzymalska K., Skopinska-Wisniewska J., Kaczmarek H., Marszałł M.P. (2017). Chitosan–collagen coated magnetic nanoparticles for lipase immobilization—new type of “enzyme friendly” polymer shell crosslinking with squaric acid. Catalysts.

[193] Sikora A., Chełminiak-Dudkiewicz D., Siódmiak T., Tarczykowska A., Sroka W.D., Ziegler-Borowska M., Marszałł M.P. (2016). Enantioselective acetylation of (R,S)-atenolol: The use of Candida rugosa lipases immobilized onto magnetic chitosan nanoparticles in enzyme-catalyzed biotransformation. J. Mol. Catal. B Enzym..

[194] Hosseini S.M., Kim S.M., Sayed M., Younesi H., Bahramifar N., Park J.H., Pyo S.H. (2019). Lipase-immobilized chitosan-crosslinked magnetic nanoparticle as a biocatalyst for ring opening esterification of itaconic anhydride. Biochem. Eng. J..

[195] Kuo C.H., Liu Y.C., Chang C.M.J., Chen J.H., Chang C., Shieh C.J. (2012). Optimum conditions for lipase immobilization on chitosan-coated Fe_3_O_4_ nanoparticles. Carbohydr. Polym..

[196] Wang X.Y., Jiang X.P., Li Y., Zeng S., Zhang Y.W. (2015). Preparation Fe_3_O_4_@chitosan magnetic particles for covalent immobilization of lipase from Thermomyces lanuginosus. Int. J. Biol. Macromol..

[197] Monteiro R.R.C., Lima P.J.M., Pinheiro B.B., Freire T.M., Dutra L.M.U., Fechine P.B.A., Gonçalves L.R.B., de Souza M.C.M., Dos Santos J.C.S., Fernandez-Lafuente R. (2019). Immobilization of lipase a from Candida antarctica onto Chitosan-coated magnetic nanoparticles. Int. J. Mol. Sci..

[198] Ziegler-Borowska M., Chełminiak D., Siódmiak T., Sikora A., Marszałł M.P., Kaczmarek H. (2014). Synthesis of new chitosan coated magnetic nanoparticles with surface modified with long-distanced amino groups as a support for bioligands binding. Mater. Lett..

[199] Ziegler-Borowska M., Chełminiak D., Kaczmarek H., Kaczmarek-Kędziera A. (2016). Effect of side substituents on thermal stability of the modified chitosan and its nanocomposites with magnetite. J. Therm. Anal. Calorim..

[200] Yi S.S., Noh J.M., Lee Y.S. (2009). Amino acid modified chitosan beads: Improved polymer supports for immobilization of lipase from Candida rugosa. J. Mol. Catal. B Enzym..

[201] Sikora A., Chełminiak-Dudkiewicz D., Ziegler-Borowska M., Marszałł M.P. (2017). Enantioseparation of (RS)-atenolol with the use of lipases immobilized onto new-synthesized magnetic nanoparticles. Tetrahedron Asymmetry.

[202] Marszałł M.P., Sroka W.D., Sikora A., Chełminiak D., Ziegler-Borowska M., Siódmiak T., Moaddel R. (2016). Ligand fishing using new chitosan based functionalized Androgen Receptor magnetic particles. J. Pharm. Biomed. Anal..

[203] Zamora-Mora V., Soares P., Echeverria C., Hernández R., Mijangos C. (2015). Composite Chitosan/Agarose Ferrogels for Potential Applications in Magnetic Hyperthermia. Gels.

[204] Gu Y., Cheong K.L., Du H. (2017). Modification and comparison of three Gracilaria spp. agarose with methylation for promotion of its gelling properties. Chem. Cent. J..

[205] Adivi F.G., Hashemi P. (2018). Ultrafine agarose-coated superparamagnetic iron oxide nanoparticles (AC-SPIONs): A promising sorbent for drug delivery applications. J. Iran. Chem. Soc..

[206] Adivi F.G., Hashemi P., Tehrani A.D. (2019). Agarose-coated Fe_3_O_4_ @SiO_2_ magnetic nanoparticles modified with sodium dodecyl sulfate, a new promising sorbent for fast adsorption/desorption of cationic drugs. Polym. Bull..

[207] Liao J., Huang H. (2020). Review on Magnetic Natural Polymer Constructed Hydrogels as Vehicles for Drug Delivery. Biomacromolecules.

[208] Chen T., Yang W., Guo Y., Yuan R., Xu L., Yan Y. (2014). Enhancing catalytic performance of β-glucosidase via immobilization on metal ions chelated magnetic nanoparticles. Enzyme Microb. Technol..

[209] Klimaviciute R., Bendoraitiene J., Lekniute E., Zemaitaitis A. (2014). Non-stoichiometric complexes of cationic starch and 4-sulfophthalic acid and their flocculation efficiency. Colloids Surfaces A Physicochem. Eng. Asp..

[210] Uthaman S., Lee S.J., Cherukula K., Cho C.S., Park I.K. (2015). Polysaccharide-coated magnetic nanoparticles for imaging and gene therapy. Biomed Res. Int..

[211] Adak S., Banerjee R. (2016). A green approach for starch modification: Esterification by lipase and novel imidazolium surfactant. Carbohydr. Polym..

[212] Ziegler-Borowska M. (2019). Magnetic nanoparticles coated with aminated starch for HSA immobilization- simple and fast polymer surface functionalization. Int. J. Biol. Macromol..

[213] Saikia C., Hussain A., Ramteke A., Sharma H.K., Deb P., Maji T.K. (2015). Carboxymethyl starch-coated iron oxide magnetic nanoparticles: A potential drug delivery system for isoniazid. Iran. Polym. J..

[214] Dung T.T., Danh T.M., Hoa L.T.M., Chien D.M., Duc N.H. (2009). Structural and magnetic properties of starch-coated magnetite nanoparticles. J. Exp. Nanosci..

[215] Zheng M., Lu J., Zhao D. (2018). Effects of starch-coating of magnetite nanoparticles on cellular uptake, toxicity and gene expression profiles in adult zebrafish. Sci. Total Environ..

[216] Wang B., Cheng F., Lu Y., Ge W., Zhang M., Yue B. (2013). Immobilization of pectinase from Penicillium oxalicum F67 onto magnetic cornstarch microspheres: Characterization and application in juice production. J. Mol. Catal. B Enzym..

[217] Gagnon P., Toh P., Lee J. (2014). High productivity purification of immunoglobulin G monoclonal antibodies on starch-coated magnetic nanoparticles by steric exclusion of polyethylene glycol. J. Chromatogr. A.

[218] Lu W., Shen Y., Xie A., Zhang W. (2013). Preparation and protein immobilization of magnetic dialdehyde starch nanoparticles. J. Phys. Chem. B.

[219] Kuroiwa T., Noguchi Y., Nakajima M., Sato S., Mukataka S., Ichikawa S. (2008). Production of chitosan oligosaccharides using chitosanase immobilized on amylose-coated magnetic nanoparticles. Process Biochem..

[220] Eyley S., Thielemans W. (2014). Surface modification of cellulose nanocrystals. Nanoscale.

[221] Habibi Y., Lucia L.A., Rojas O.J. (2010). Cellulose nanocrystals: Chemistry, self-assembly, and applications. Chem. Rev..

[222] Van Rie J., Thielemans W. (2017). Cellulose-gold nanoparticle hybrid materials. Nanoscale.

[223] Aguilera G., Berry C.C., West R.M., Gonzalez-Monterrubio E., Angulo-Molina A., Arias-Carrión Ó., Méndez-Rojas M.Á. (2019). Carboxymethyl cellulose coated magnetic nanoparticles transport across a human lung microvascular endothelial cell model of the blood-brain barrier. Nanoscale Adv..

[224] Namdeo M., Bajpai S.K. (2009). Immobilization of α-amylase onto cellulose-coated magnetite (CCM) nanoparticles and preliminary starch degradation study. J. Mol. Catal. B Enzym..

[225] Ivanova V., Petrova P., Hristov J. (2011). Application in the Ethanol Fermentation of Immobilized Yeast Cells in Matrix of Alginate/Magnetic Nanoparticles, on Chitosan-Magnetite Microparticles and Cellulose-coated Magnetic Nanoparticles. arXiv.

[226] Anirudhan T.S., Rejeena S.R., Binusree J. (2013). Adsorptive separation of myoglobin from aqueous solutions using iron oxide magnetic nanoparticles modified with functionalized nanocrystalline cellulose. J. Chem. Eng. Data.

[227] Zhang J., Feng X., Wang J., Fang G., Liu J., Wang S. (2020). Nano-crystalline cellulose-coated magnetic nanoparticles for affinity adsorption of glycoproteins. Analyst.

[228] Mohammadi F., Moeeni M., Li C., Boukherroub R., Szunerits S. (2020). Interaction of cellulose and nitrodopamine coated superparamagnetic iron oxide nanoparticles with alpha-lactalbumin. RSC Adv..

[229] Ohannesian N., De Leo C.T., Martirosyan K.S. (2019). Dextran coated superparamagnetic iron oxide nanoparticles produced by microfluidic process. Mater. Today Proc..

[230] Unterweger H., Dézsi L., Matuszak J., Janko C., Poettler M., Jordan J., Bäuerle T., Szebeni J., Fey T., Boccaccini A.R. (2018). Dextran-coated superparamagnetic iron oxide nanoparticles for magnetic resonance imaging: Evaluation of size-dependent imaging properties, storage stability and safety. Int. J. Nanomed..

[231] Naha P.C., Liu Y., Hwang G., Huang Y., Gubara S., Jonnakuti V., Simon-Soro A., Kim D., Gao L., Koo H. (2019). Dextran-Coated Iron Oxide Nanoparticles as Biomimetic Catalysts for Localized and pH-Activated Biofilm Disruption. ACS Nano.

[232] Hradil J., Pisarev A., Babič M., Horák D. (2007). Dextran-modified iron oxide nanoparticles. China Particuology.

[233] Bai H., Liu Z., Sun D.D. (2011). Highly water soluble and recovered dextran coated fe3o 4 magnetic nanoparticles for brackish water desalination. Sep. Purif. Technol..

[234] Vasić K., Knez Ž., Konstantinova E.A., Kokorin A.I., Gyergyek S., Leitgeb M. (2020). Structural and magnetic characteristics of carboxymethyl dextran coated magnetic nanoparticles: From characterization to immobilization application. React. Funct. Polym..

[235] Yu M.K., Park J., Jon S. (2012). Targeting strategies for multifunctional nanoparticles in cancer imaging and therapy. Theranostics.

[236] Weissleder R., Moore A., Mahmood U., Bhorade R., Benveniste H., Chiocca E.A., Basilion J.P. (2000). In vivo magnetic resonance imaging of transgene expression. Nat. Med..

[237] Hogemann D., Josephson L., Weissleder R., Basilion J.P. (2000). Improvement of MRI probes to allow efficient detection of gene expression. Bioconjug. Chem..

[238] Horng H.E., Yang S.Y., Hong C.Y., Liu C.M., Tsai P.S., Yang H.C., Wu C.C. (2006). Biofunctionalized magnetic nanoparticles for high-sensitivity immunomagnetic detection of human C-reactive protein. Appl. Phys. Lett..

[239] Templin M.F., Stoll D., Schrenk M., Traub P.C., Vöhringer C.F., Joos T.O. (2002). Protein microarray technology. Trends Biotechnol..

[240] Li J., Zhou Y., Li M., Xia N., Huang Q., Do H., Liu Y.N., Zhou F. (2011). Carboxymethylated dextran-coated magnetic iron oxide nanoparticles for regenerable bioseparation. J. Nanosci. Nanotechnol..

[241] Ziv O., Avtalion R.R., Margel S. (2008). Immunogenicity of bioactive magnetic nanoparticles: Natural and acquired antibodies. J. Biomed. Mater. Res. Part A.

[242] Narain R., Gonzales M., Hoffman A.S., Stayton P.S., Krishnan K.M. (2007). Synthesis of monodisperse biotinylated p(NIPAAm)-coated iron oxide magnetic nanoparticles and their bioconjugation to streptavidin. Langmuir.

